# Synergistic SERS Enhancement in GaN‐Ag Hybrid System toward Label‐Free and Multiplexed Detection of Antibiotics in Aqueous Solutions

**DOI:** 10.1002/advs.202100640

**Published:** 2021-08-07

**Authors:** Kang Hyun Lee, Hanhwi Jang, Yoon Seok Kim, Chul‐Ho Lee, Seunghee H. Cho, Minjoon Kim, Hoki Son, Kang Bin Bae, Dung Van Dao, Yeon Sik Jung, In‐Hwan Lee

**Affiliations:** ^1^ Department of Semiconductor Systems Engineering Korea University 145 Anam‐ro, Seongbuk‐gu Seoul 02841 Republic of Korea; ^2^ Department of Materials Science and Engineering Korea Advanced Institute of Science and Technology (KAIST) 291 Daehak‐ro, Yuseong‐gu Daejeon 34141 Republic of Korea; ^3^ KU‐KIST Graduate School of Converging Science and Technology Korea University 145 Anam‐ro, Seongbuk‐gu Seoul 02841 Republic of Korea; ^4^ Department of Integrative Energy Engineering Korea University 145 Anam‐ro, Seongbuk‐gu Seoul 02841 Republic of Korea; ^5^ Department of Materials Science and Engineering Korea University 145 Anam‐ro, Seongbuk‐gu Seoul 02841 Republic of Korea

**Keywords:** antibiotics, gallium nitride nanopillars, multiplexing, surface‐enhanced Raman spectroscopy, silver nanowires

## Abstract

Noble metal‐based surface‐enhanced Raman spectroscopy (SERS) has enabled the simple and efficient detection of trace‐amount molecules via significant electromagnetic enhancements at hot spots. However, the small Raman cross‐section of various analytes forces the use of a Raman reporter for specific surface functionalization, which is time‐consuming and limited to low‐molecular‐weight analytes. To tackle these issues, a hybrid SERS substrate utilizing Ag as plasmonic structures and GaN as charge transfer enhancement centers is presented. By the conformal printing of Ag nanowires onto GaN nanopillars, a highly sensitive SERS substrate with excellent uniformity can be fabricated. As a result, remarkable SERS performance with a substrate enhancement factor of 1.4 × 10^11^ at 10 fM for rhodamine 6G molecules with minimal spot variations can be realized. Furthermore, quantification and multiplexing capabilities without surface treatments are demonstrated by detecting harmful antibiotics in aqueous solutions. This work paves the way for the development of a highly sensitive SERS substrate by constructing complex metal‐semiconductor architectures.

## Graphical abstract

The synergistic interplay of the electromagnetic and chemical enhancement of Raman signals is achieved in an Ag nanowire/GaN nanopillar‐based hybrid SERS structure to maximize signal intensities. Using the solvent‐assisted nanotransfer printing technique, highly ordered metal nanostructures are fabricated conformally on a nanopatterned semiconductor substrate. Therefore, several antibiotics dissolved in aqueous solutions are detected without any Raman reporters.

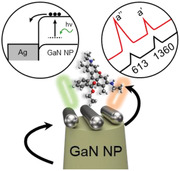

## 1 Introduction

A surface‐enhanced Raman spectroscopy (SERS) is a highly functional and practicable analytical technology with extremely high sensitivity (a very low limit of detection (LOD)) that is capable of exponentially enhancing Raman intensity.^[^ ^1^ ^]^ Its highly sensitive identification capability for detecting molecules enables its application in diverse disciplines, such as chemistry, biology, medicine, pharmacology, forensic, environmental science, and toxin detection.^[^ ^2^ ^]^ Previous research has focused on the development of electromagnetic mechanism (EM)‐based SERS substrates. The EM enhancement describes the enhanced local electromagnetic fields in “hot spots” where localized surface plasmon resonance is excited by the incident light in plasmonic nanostructures.^[^ ^3^ ^]^ Noble metals, such as Ag, Au, and Cu, have been widely exploited as materials for plasmonic nanostructures based on their high substrate enhancement factor (SEF), a reference measure of SERS performance on solid analytes,^[^ ^4^ ^]^ at the order of 10^6^–10^10^ (Table S5, Supporting Information) due to their formidable contribution of EM.^[^ ^2^, ^5^ ^]^ For example, Lim et al. designed Au nanobridged particles that have a 1 nm interior gap showing a maximum SEF of 5 × 10^9^.^[^ ^6^ ^]^ However, in many cases, highly complicated synthesis procedures often raise challenges regarding large‐scale fabrication and cost‐effectiveness. Furthermore, metal nanostructures are often destabilized by significant photothermal effects under laser excitation.^[^ ^7^ ^]^

To deal with the aforementioned issue, we previously proposed a 3D‐stacked cross‐point nanonetwork by a solvent‐assisted nanotransfer printing (S‐nTP) method.^[^ ^8^ ^]^ Utilizing S‐nTP to fabricate an SERS substrate provided low‐cost macroscopic reproducibility. By sequential printing of Ag nanowires on an Ag film, an SEF of 4 × 10^7^ was obtained.^[^ ^8^ ^]^ More recently, we also reported cross‐point nanowelding of Au nanowires and aptamer‐functionalization to enable the multiplexing of analytes at pM levels.^[^ ^9^ ^]^ Appropriate surface functionalization on plasmonic nanostructures can achieve target specificity and improve the detection limit.^[^ ^10^ ^]^ However, to more reliably detect trace‐level target molecules with a small Raman cross‐section, an SERS substrate with an intrinsically high SEF and uniformity should be developed.

Unlike EM, the chemical mechanism (CM) originates from charge transfer resonance, usually in semiconductors.^[^ ^3^, ^11^ ^]^ Typical examples of CM‐based SERS include MoS_2_, Cu_2_O, Ta_2_O_5_, and GaN.^[^ ^2^, ^12^ ^]^ These materials offer both uniform SERS signals and better chemical stability.^[^ ^5a^ ^]^ However, the SEFs (usually in the range 10^1^–10^5^) of CM‐based SERS substrates are far less than those of their noble‐metal counterparts (Table S6, Supporting Information).^[^ ^12^, ^13^ ^]^ While a hybrid SERS substrate was developed using GaN and Ag to improve the low sensitivity of semiconductor‐based SERS, this only showed a limited SEF of 10^5^.^[^ ^14^ ^]^ In addition, most of hybrid SERS substrates have exhibited slightly higher or even lower SEF values compared to those of metals, which significantly limits a broad application of hybrid systems to the SERS analytics (Table S7, Supporting Information). This implies that the simple decoration of semiconductors with plasmonic metals cannot significantly improve SERS performance and requires more sophisticated design rules.

Herein, we propose a strategy to synergistically improve an SERS performance via Ag‐GaN hybrid nanostructures. With an extensive study on the effects of substrate morphologies and plasmonic nanostructures, we present an optimized hybrid structure using vertically oriented GaN nanopillars (NPs) and Ag nanowires (NWs). GaN NPs act as a charge transfer enhancement center with substantially increased surface area compared to that of a GaN epilayer. By utilizing the S‐nTP technique, highly ordered and uniformly oriented Ag NWs are conformally printed on GaN NPs to provide strong EM enhancement from plasmonic nanostructures. As a result, we could fabricate a highly sensitive SERS substrate with a remarkable SERS SEF of 1.4 × 10^11^. By using quantitative SERS intensity analysis, we corroborate the co‐existence of both EM and CM enhancement in the fabricated SERS substrate. Moreover, we also demonstrate label‐free quantification and the multiplexing of antibiotics dissolved in an aqueous solution enabled by improved SERS performance.

## 2 Results and Discussion

### 2.1 Fabrication of the AoG Substrate

GaN has been widely exploited as a material for fabricating a wide range of nanostructures such as NPs, nanopits, and nano‐pyramids by various etching methods. In particular, among the diverse types of GaN nanostructures, we selected the NPs due to their high SEF for SERS.^[^ ^12c^ ^]^ The control of etching time, etching mode, and etchant sources can modulate the size, density, and interval of GaN NPs, which has a substantial impact on the formation of SERS hot spots, areas in which local electromagnetic fields are greatly amplified.^[^ ^12c^ ^]^

**Figure 1:**
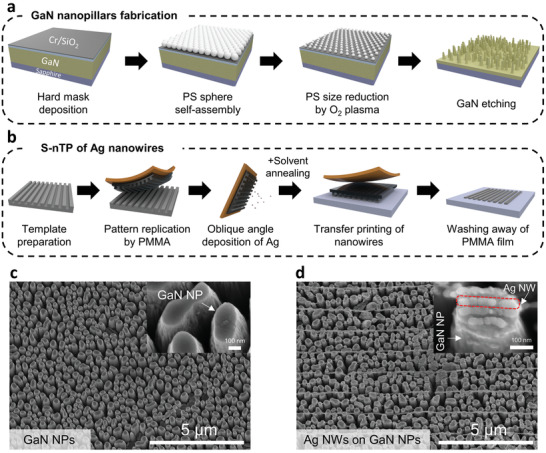
Fabrication process of Ag NWs on GaN NPs (AoG) structure. Schematic drawing of the fabrication process of a) GaN NPs and b) Ag NWs. Typical SEM images of c) GaN NPs and d) AoG structure with a tilting angle of 50°. The inset in each image is a high‐magnification image of NPs in (c) and (d).

**Figure** **1a** shows the process for fabricating the NP array by polystyrene (PS) nanosphere lithography (NSL). After the deposition of a Cr/SiO_2_ etch mask, hexagonally close‐packed PS spheres were transferred from the surface of the water to the GaN epilayer. The role of the PS spheres is to transfer hexagonal patterns to the Cr/SiO_2_ hard mask. The diameter of the PS nanospheres was then reduced by adjusting the etching time via reactive ion etching (RIE). These PS nanospheres formed highly uniform and periodic assembly structures across the whole wafer surface. While PS nanospheres cannot provide sufficient etch resistance to fabricate high‐aspect‐ratio patterns, the Cr/SiO_2_ layer was employed as an excellent hard mask to deeply etch down the GaN epilayer. After patterning a Cr and SiO_2_ hard mask using PS nanosphere etch masks, GaN was etched via inductively coupled plasma‐reactive ion etching (ICP‐RIE). Figure 1c shows a typical SEM image of fabricated GaN NPs. Each singular NP has an average length of 2 µm and a diameter in the range 200–300 nm.

To prepare the AoG (Ag NWs on GaN NPs) structure using the fabricated GaN NPs, we utilized solvent‐assisted nanotransfer printing (S‐nTP).^[^ ^8^, ^15^ ^]^ Previous studies have shown that S‐nTP can fabricate ultrahigh‐resolution patterns with excellent uniformity conformally on an arbitrary surface.^[^ ^16^ ^]^ Figure 1b shows a schematic drawing illustrating the S‐nTP process. A pattern of the Si master template prepared by directed self‐assembly of a block copolymer (BCP) is replicated with a polymethylmethacrylate (PMMA) layer (Figure S13, Supporting Information). After detaching the PMMA replica from the master template using polyimide (PI) adhesive tape, Ag NWs were formed by the oblique‐angle deposition of Ag via e‐beam evaporation, followed by contacting the Ag NWs on PMMA/PI on the GaN NPs. After exposing the solvent vapor for a few seconds, PMMA was released from the adhesive tape due to adhesion weakening at the PMMA/adhesive interface.^[^ ^15^ ^]^ By washing the PMMA with toluene, highly ordered Ag NWs were physically attached to the receiver substrate.

Previous research using S‐nTP typically fabricated plasmonic nanostructures on a flat substrate.^[^ ^9^, ^17^ ^]^ However, due to the highly conformal printing capability of S‐nTP, we could fabricate Ag NWs with sub‐20 nm width on the top surface of GaN NPs (Figure 1d). Another potential route for fabricating similar structures is to drop‐cast solution‐processed Ag NWs on GaN NPs. However, solution‐processed Ag NWs are usually passivated with an organic surfactant, which hinders the facile charge transfer between metals and analyte molecules.^[^ ^18^ ^]^ Moreover, the precise control of the NW dimension is challenging, particularly for narrow NWs.^[^ ^19^ ^]^ Therefore, Ag NWs fabricated by S‐nTP offer several advantages in terms of charge conduction properties and size uniformity compared to synthesized Ag NWs.

### 2.2 Structure‐Dependent SERS Performance

**Figure 2:**
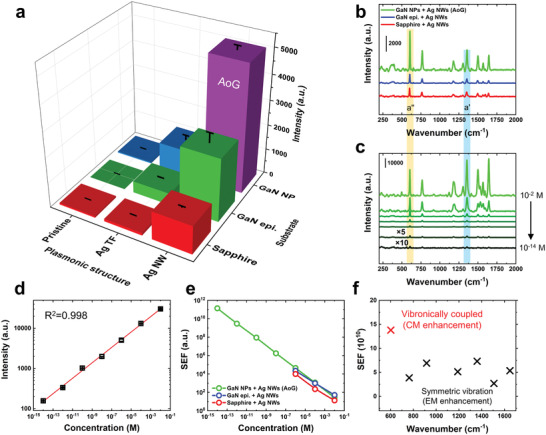
SERS enhancement of the AoG structure. a) Comparison of the Raman intensities of R6G (10^−6^ m) absorbed on various substrates. b) SERS spectra of R6G (10^−6^ m) acquired on substrates with Ag NWs as a plasmonic structure. c) Concentration‐dependent SERS spectra of R6G absorbed on the AoG structure. Orange and blue regions in (b) and (c) denote wavenumbers for asymmetric (a″) and symmetric (a′) vibrations, respectively. d) Standard curve for R6G using an intensity at 613 cm^−1^ in (c). Error bar denotes the standard deviation of SERS intensity, which was calculated from five individual measurements. e) Calculated SEF using an intensity at 613 cm^−1^ for substrates with Ag NWs. f) Calculated SEF of the AoG structure at various wavenumbers.

**Figure** **2a** compares the SERS intensities of nine SERS samples at 10^−6^ m R6G. In terms of the effect of the substrate on the Raman intensity, the degree of enhancement increased in the order of sapphire, GaN epilayer, and GaN NP. This can be attributed to the EM enhancement that occurs when the GaN epilayer is fabricated in the form of pillars and to the CM enhancement (charge transfer) that occurs in GaN and not in sapphire.^[^ ^20^ ^]^ In terms of the effect of the plasmonic structure on Raman intensity, the degree of enhancement was elevated in the order of pristine, Ag thin film (Ag TF), and Ag NWs due to the EM enhancement resulting from Ag nanostructures.^[^ ^8^ ^]^ Although the SERS intensities of Ag NWs are relatively stronger than that of Ag TF, the Ag TF/GaN epilayer also showed high sensitivity, which may originate from the rough surface morphologies and nano‐sized grains of deposited Ag films (Figure S1, Supporting Information). As shown in Figure 2a, the intensity of the AoG (the purple bar) is stronger than that of the Ag TF/GaN epilayer system (the bar in the center), which can be attributed to the EM effects resulting from the nanoscale geometries of Ag NWs and GaN NPs. See Figure S10, Supporting Information for concentration‐dependent SERS spectra of nine SERS substrates.

Figure 2b shows that AoG has a greater SERS effect than other substrates based on Ag NWs; in particular, the strongest enhancement is seen at wavenumber 613 cm^−1^ among all wavenumbers. It should be noted that the Raman scattering of R6G at 613 cm^−1^ stems from asymmetric (a″) vibration, while the peaks at other wavenumbers are symmetric vibrations (a′). As shown in Figure 2c, as the molar concentration of R6G decreases, the peak intensities of R6G also decreases, but the characteristic peak is detected up to 10^−14^ m. Interestingly, the intensity ratios of the a′ to a″ modes decrease as the concentration decreases. This observation supports that the charge transfer mechanism is particularly significant at low concentrations where analyte molecules have less opportunity to be located near hot spots.^[^ ^21^ ^]^ Finite‐difference time‐domain (FDTD) simulation results show that strong hot spots are formed near the gaps between the Ag NWs and GaN NPs with an approximate width of 5 nm (Figure S2, Supporting Information). Therefore, analytes are less likely to be located near hot spots at extremely low concentrations, which only cover a small area of the substrate. Moreover, an LOD of 10^−14^ m and extremely low standard deviations among signals were confirmed through repeated measurements (Figure 2d). This is due to S‐nTP forming excellently ordered Ag NWs onto GaN NPs, which enables high reproducibility and high uniformity. A three‐sigma test of 10^−14^ m spectrum further supports that the peaks clearly result from analyte signals (Figure S3, Supporting Information). Furthermore, spectra acquired from 10 random spots as well as SERS mapping were extremely uniform and reproducible (Figures S4 and S16, Supporting Information).

Figure 2e indicates that the SEF values of other SERS substrates (Ag NWs on sapphire and GaN epilayer) are lower than those of AoG, which can be attributed to the increased hydrophobicity of the substrate due to the nanostructured surface known as the Cassie–Baxter state.^[^ ^22^ ^]^ It was reported that a textured surface is beneficial for improving SERS signals by concentrating the analyte molecules.^[^ ^23^ ^]^ Similarly, we observed that both GaN NPs and Ag NWs contribute to the increase of the water contact angle (Figure S5, Supporting Information), which can be beneficial for focusing analytes on local spots, particularly at extremely low concentrations. This could be attributed to the trapping of the analyte molecules in the gaps of Ag NWs and GaN NPs during drying.

**Figure 3:**
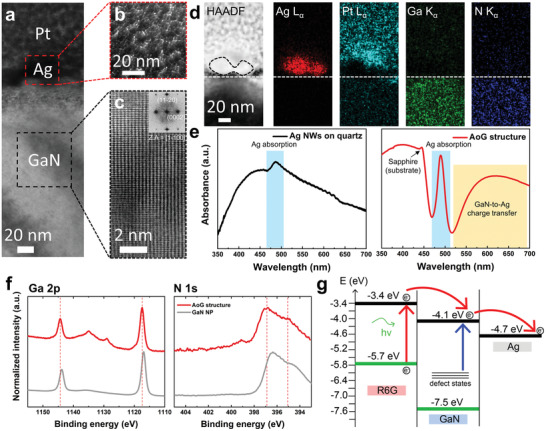
Structural, optical, and physiochemical characterization of the AoG structure. a) Low‐magnification TEM image of the AoG structure and HRTEM image of b) Ag NW and c) GaN substrate. The inset is the fast Fourier transform (FFT) image of (c). d) HAADF‐STEM image and corresponding EDS elemental mapping of the AoG structure. The white dotted line denotes the interface between Ag NW and the GaN substrate. e) Optical absorption spectrum of Ag NW on quartz (black) and the AoG structure (red). f) XPS spectra of GaN NP and the AoG structure. Both the Ga 2p and N 1s orbitals of the AoG structure show a peak shift to a higher binding energy compared to that of GaN NP. g) Schematic energy‐level diagram of R6G on the AoG structure with respect to the vacuum level.

In addition, more surface defect states are formed due to the increased surface dangling bonds of the GaN NPs compared to those of the GaN epilayer. These defect states would lower the charge transfer barrier between GaN and Ag.^[^ ^24^ ^]^ Overall, in the AoG structure, a remarkable SEF of 1.4 × 10^11^ can be obtained due to the formation of more hot spots by nanometer‐sized gaps (between GaN NPs, Ag NWs, and between the two, as presented in Figures 1d and 3d) and charge transfer occurs between the analyte and the NP substrate, in addition to the increased hydrophobicity at the surface. For these reasons, the AoG system with an LOD of 10^−14^ m and an SEF of 1.4 × 10^11^ had the most remarkable SERS performance among other hybrid SERS systems, for example, nanoporous Au/SnO/Ag (1.0 × 10^10^),^[^ ^25^ ^]^ Ag/Ge (1.3 × 10^9^),^[^ ^26^ ^]^ Au@Ag/Si (1.2 × 10^9^),^[^ ^27^ ^]^ and Ag/graphene oxide (7.0 × 10^8^).^[^ ^28^ ^]^ Two commonly utilized SERS performance indicators—1) analytical enhancement factor (AEF) and 2) substrate enhancement factor (SEF) need to be distinguished for a more objective comparison. Due to their different definitions, AEF should be applied for dispersions and liquid SERS‐active colloids, while SEF is for solid substrates and nanostructured interfaces in a dry state. Therefore, we only compared the enhancement factor values that followed the calculation protocols of SEF. Comparison of SEF of the AoG substrate with other state‐of‐the‐art SERS substrates is also provided in Figure S12, Supporting Information.^[^ ^3c^ ^]^

It is well known that surface‐enhanced resonant Raman scattering (SERRS) occurs when the laser wavelength is similar to the absorption peak of the analyte molecule.^[^ ^29^ ^]^ As R6G strongly absorbs light with a wavelength of 532 nm, its increased Raman cross‐section results in strong enhancements of Raman intensity and eventually reduces the achievable detection limit. To compare the effect of molecular resonance on LOD, we measured the SERS spectra of additional Raman reporters (malachite green, 4‐mercaptopyridine, and 4‐mercaptobenzoic acid) that have various absorption peaks (Figure S6, Supporting Information). It was observed that the LOD of these analytes was 10^−12^ m under 532 nm excitation, which is slightly higher than that of R6G due to a mismatch between the laser wavelength and the absorption peak. However, the asymmetric vibration mode peak was more enhanced than those of the symmetric vibration modes, particularly for low concentrations (10^−6^–10^−12^ m), which is consistent with the tendency obtained with R6G. In addition, we observed that the LOD of malachite green can be reduced to as low as 10^−14^ m when using a 633 nm laser (Figure S7, Supporting Information). These results corroborate the robustness of the AoG substrate for a wide range of target concentrations, as well as the improved signal enhancement under the SERRS condition.

As shown in Figure 2f, the SEF calculated at 613 cm^−1^ is almost threefold compared to that calculated at other wavenumbers such as 772, 917, 1187, 1361, 1510, and 1648 cm^−1^. This implies that the charge transfer mechanism exists in the AoG substrate. According to the Herzberg–Teller selection rule, the vibration mode of the absorbed molecule would be enhanced when it is vibronically coupled.^[^ ^12b^ ^]^ The chemical enhancement from the molecule–semiconductor charge transfer is given by the following expression:

(1) $$\begin{array}{ccc} & & R_{ICK}\left(\omega \right)\\ & & =\frac{\mu_{KI}\mu_{IC}h_{CK}\left\langle i\right|Q_{k}\left|f\right\rangle }{\left({\left(\varepsilon_{1}\left(\omega \right)\;+\;2\varepsilon_{0}\right)}^{2}\;+\;\varepsilon_{2}^{2}\left(\omega \right))((\omega_{IC}^{2}\;-\;\omega^{2})\;+\;\gamma_{IC}^{2})((\omega_{KI}^{2}\;-\;\omega^{2})\;+\;\gamma_{KI}^{2}\right)} \end{array}$$

where subscripts I, K, V, and C denote the HOMO, LUMO, valence band maximum, and the conduction band minimum levels, respectively.^[^ ^30^ ^]^ In the case of SERRS, the charge transfer process “borrows” the intensity by coupling with the molecular transitions (at *ω* = *ω*_*IK*_) associated with the Herzberg–Teller coupling term (*h_CK_*). For the signal enhancement, all terms in the numerator 〈*i*|*Q_k_* |*f*〉, *μ*_*KI*_, *μ*_*IC*_, and *h_CK_* must be non‐zero. This leads to the following selection rule^[^ ^31^ ^]^

(2) $$\Gamma \left(Q_{K}\right)=\Gamma \left(\mu_{CT}\right)\times \Gamma \left(\mu_{mol}\right)$$

where Γ(*Q_k_*) is the irreducible representation to which the allowed SERS vibration belongs, while Γ(*μ*_*CT*_) and Γ(*μ*_*mol*_) are those of the charge transfer transition and molecular transition where the intensity is borrowed.^[^ ^30b^ ^]^ In the case of R6G, only out‐of‐plane vibrations (a″) will be selectively amplified because Γ(*μ*_*CT*_) is perpendicular to the molecular symmetry plane.^[^ ^32^ ^]^ The vibration of R6G at 613 cm^−1^ corresponds to the out‐of‐plane bending motion of carbon atoms in the xanthene skeleton, which is an asymmetric vibration and vibronically coupled to the charge transfer enhancement (Figure S8, Supporting Information and Table S2, Supporting Information).^[^ ^2f^ ^]^

### 2.3 Characterization of the AoG Structure

To gain deeper insights into the enhancement mechanism of the AoG structure, we conducted extensive characterizations to identify structural, optical, and physiochemical properties of the AoG structure. **Figure** **3a** shows a low‐magnification TEM image of the AoG structure; it shows that a 20‐nm‐wide Ag NW contacts conformally onto the GaN substrate. From the high‐resolution TEM (HRTEM) image in Figure 3b, one can note that the grain size of the Ag NW is typically tens of nanometers, showing a general polycrystalline feature of e‐beam deposited metals.^[^ ^33^ ^]^ In contrast, the GaN substrate shows long‐range order crystallinity, as shown in Figure 3c. From the fast Fourier transform (FFT) image in (c), the zone axis is indexed to [1‐100], which is normal to the [0001] direction. Figure 3d shows a high‐angle annular dark field‐scanning transmission electron microscopy (HAADF‐STEM) image and the corresponding STEM‐EDS elemental mapping of the AoG structure. EDS mapping data confirm that Ag NWs are well‐formed onto the GaN substrate without severe structural deformation (see Figures S14 and S15, Supporting Information for the additional SEM‐ and TEM‐EDS characterizations).

We then compare the optical characteristics of the AoG structure to Ag NWs printed on a quartz substrate. Figure 3e shows the optical absorption spectra of Ag NWs printed on the quartz substrate and GaN NPs, respectively. Although both spectra have a distinct absorption peak near 490 nm, which arises from the plasmon absorption of Ag NWs,^[^ ^18b^ ^]^ the absorption was substantially intensified by the existence of GaN. Additionally, a broad absorption peak was observed in the near‐IR region of the AoG structure. These results suggest that electron transfer occurs from GaN to Ag NWs in the AoG structure, which is consistent with a previous study that reported that electron transfer from CdSe quantum dots to Au nanoparticles increases optical absorption near 650 nm.^[^ ^34^ ^]^ This is also supported by a previous study, which reported charge transfer from GaN to Ag and a resultant increase of the surface charge density of Ag.^[^ ^35^ ^]^ Our X‐ray photoelectron spectroscopy (XPS) analysis results in Figure 3f further corroborate the electron transfer between GaN and Ag. The XPS peaks for the Ga 2p and N 1s orbitals of the AoG structure shift toward a higher binding energy level compared to that of bare GaN NP. This implies a loss of electrons in a GaN system upon contact with Ag NWs.

Based on these observations, we can speculate that the high SERS performance capabilities of the AoG structure may originate from the CM enhancement combined with the EM enhancement. Ag NWs provide a significant enhancement of the SERS signal due to plasmonic hot spots close to target molecules.^[^ ^36^ ^]^ Nevertheless, the EM enhancement alone is not sufficient for explaining significant signal enhancement and the ultra‐low detection limit of 10^−14^ m, as confirmed in Figure 2c. As discussed above, the high surface energy of AoG can concentrate analyte molecules, and lead to improved signal intensities. Moreover, high‐aspect‐ratio protrusions in GaN NPs would divide Ag NWs into several Ag nanorods, in contrast to the morphology of Ag NWs printed on a GaN epilayer (Figure S9, Supporting Information). This would be beneficial for forming a nano‐sized gap that would maximize the EM enhancement. In addition, the exposed sidewalls of GaN NPs can provide a facile charge transport route through a molecule–semiconductor–metal pathway, as shown in the schematic energy‐level diagram of R6G on the AoG substrate in Figure 3g. The highest occupied molecular orbital (HOMO) and the lowest unoccupied molecular orbital level (LUMO) of R6G are −5.7 and −3.4 eV, respectively.^[^ ^2f^ ^]^ The charge transfer process starts with electron excitation from the HOMO to the LUMO level of R6G by the Raman excitation laser. In addition, trapped electrons at the defect states of GaN can be excited by the photon energy. These electrons are transferred to the conduction band of GaN and finally injected to the Fermi level of Ag. Therefore, the polarizability of the R6G molecule changes and the Raman intensity is enhanced by the charge transfer mechanism.^[^ ^2^, ^37^ ^]^ Consequently, GaN‐Ag hybrid structure would be a suitable system for constructive interaction between EM and CM for maximizing SERS signals, where the charge transfer process can borrow the intensity from EM‐enhanced Raman scattering processes.

### 2.4 Label‐Free and Multiplexed SERS Detection of Antibiotics

Metronidazole (MNZ) is utilized in both humans and animals to cure diseases caused by various anaerobic bacteria and protozoa. Similar to MNZ, tetracycline (TC) has been exploited as a robust antibiotic for diverse microorganisms (e.g., chlamydia, gram‐positive, and negative bacteria, rickettsia, and protozoan parasites).^[^ ^38^ ^]^ Moreover, it has been used as a growth promoter and medicine to cure mastitis among dairy animal species during lactation.^[^ ^39^ ^]^ This may cause biomagnification by the accumulation of antibiotics in dairy products.^[^ ^40^ ^]^ In addition, these antibiotics can be harmful to human health. For example, MNZ can be carcinogenic and may damage DNA by creating reactive oxygen species.^[^ ^41^ ^]^ TC can have numerous adverse effects on consumers, for example, liver damage, gastrointestinal disorders, and allergic reactions.^[^ ^42^ ^]^

**Figure 4:**
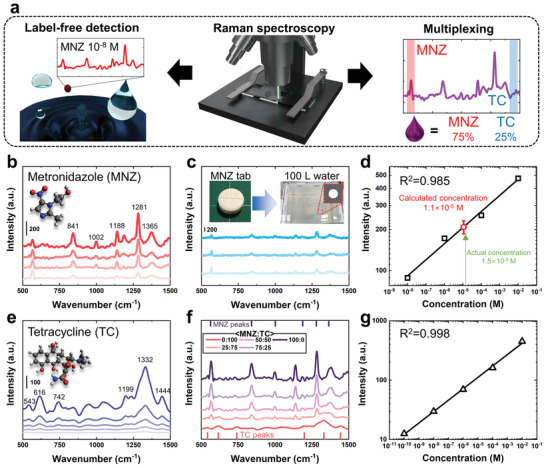
Label‐free detection and multiplexing of antibiotics in water. a) Schematic diagram showing antibiotic detection capabilities by the AoG structure and Raman spectroscopy. b) SERS spectra of MNZ from 10^−2^–10^−8^ m. c) SERS spectra of the MNZ tablet (250 mg) dissolved in 100 L of sand suspension in water. Each spectrum was acquired from three randomly selected spots. d) Standard curve drawn for MNZ derived at 1281 cm^−1^ from (b). Estimated concentration of the MNZ tablet from (c) is shown by a red circle. e) SERS spectra of TC from 10^−2^–10^−10^ m. f) Multiplexed detection of MNZ and TC of 10^−6^ m for different mixing ratios. Blue and red regions denote peaks emerging from MNZ and TC, respectively. g) Standard curve drawn for TC derived at 1332 cm^−1^ from (e).

Although the use of MNZ in food‐producing species is prohibited in the EU, the United States, and many other countries,^[^ ^41^ ^]^ a global study revealed that various antibiotics have been found in several rivers worldwide.^[^ ^43^ ^]^ Therefore, simple and efficient detection methods are needed to detect residual antibiotics in aqueous solutions. Here, we demonstrate the label‐free detection of MNZ in aqueous solutions and multiplexing MNZ and TC in various mixture solutions (**Figure** **4a**). Figure 4b,e demonstrates Raman spectra of MNZ and TC obtained for various molar concentrations, leading to LODs <10^−8^ m and <10^−10^ m for MNZ and TC, respectively. These values are far lower than those reported in previous studies,^[^ ^44^ ^]^ confirming the outstanding Raman signal amplification capability of AoG. As shown in Figure 4d,g, we could draw standard curves for MNZ and TC that have high linearity.

We next demonstrate the practical use of the AoG substrate by the quantification of MNZ from a commercial MNZ tablet (Flasinyl) in water. One Flasinyl tablet containing 2.50 × 10^−3^ g of MNZ and other additives such as corn starch, magnesium stearate, and lactose hydrate was dissolved in 100 L of tap water containing soil and sand to simulate real situations in which antibiotics are dumped in rivers or swamps. After 7 µL of tablet‐dissolved water was drop‐casted on the AoG substrate, it was dried for 1 h. Figure 4c shows the label‐free SERS spectra of the tablet solution from three random spots with high reproducibility. Assuming that the tablet becomes fully dissolved in water, the estimated concentration of MNZ will be 1.5 × 10^−5^ m. From SERS intensities at 1281 cm^−1^ and the standard curve in Figure 4d, the measured concentration is 1.1 × 10^−5^ m. Therefore, the AoG structure is capable of the accurate quantification of trace‐amount MNZ from solutions that contain various other impurities due to synergistic SERS enhancements.

Finally, we evaluate the selective detection capability of the AoG substrate via two‐component multiplexing with binary antibiotic solutions. Previous studies required reporter molecules to achieve clear multiplexing by an SERS.^[^ ^45^ ^]^ In contrast, the AoG structure allows for the expansion of the range of analytes for multiplexing due to their ability to detect the original Raman signals of analytes without any Raman reporter. Figure 4f shows the SERS spectra of antibiotic solutions (in 10^−6^ m) with five different mixing ratios of MNZ and TC (100:0, 75:25, 50:50, 25:75, 0:100). One can see that characteristic peaks from MNZ (e.g., 1139, 1281, and 1379 cm^−1^) gradually decreases as the molar fraction of TC (1199, 1332, and 1444 cm^−1^) increases, and vice versa. Therefore, antibiotic mixture solutions can be differentiated without any surface functionalization of an SERS substrate.

## 3 Conclusions

In this work, we successfully fabricated a novel‐hybrid SERS system, an AoG structure, by utilizing the interaction between extremely uniform Ag nanowires and GaN. While conventional SERS solely has relied on non‐uniform hot spots, thus sacrificing reproducibility, the AoG structure possesses ordered nanostructures and several charge transfer centers to further enhance the Raman signals with high reproducibility. This synergistic enhancement resulted in high sensitivity compared to that of other SERS systems and yielded a remarkable SEF of 1.4 × 10^11^, one of the highest values among hybrid SERS systems. In addition, this SERS‐active system achieved multiplex detection and the highly accurate quantification of antibiotics without the use of a Raman reporter or aptamers. We also showed evidence of the charge transfer between the plasmonic structure and the nanostructured substrate in a hybrid system as well as that between analyte molecules and the plasmonic structure. Furthermore, nanometer‐sized gaps that exist between Ag nanowires and an exposed surface of GaN nanopillar protrusions result in stronger electromagnetic and charge transfer enhancement, respectively. Thus, our promising and highly sensitive AoG system may provide an alternative practical SERS system for molecular differentiation and accurate quantification. A quantitative evaluation on the degree of EM and CM enhancement in a metal‐semiconductor hybrid system remains an intriguing future work.

## 4 Experimental Section

### Fabrication of Semiconductor Nanopillar Substrate

Figure 1a depicts the fabrication process of GaN NPs composed of NSL and dry etching phases. A GaN epilayer was grown in the direction of the c‐axis (0001) on a sapphire wafer via metal–organic chemical vapor deposition (MOCVD). For the purpose of fabricating a GaN NP array on a wafer scale, a 1‐µm‐thick SiO_2_ hard mask layer was deposited by PECVD (Plasmalab 800 Plus, Oxford Instruments) using 2% SiH_4_ and N_2_O on a GaN substrate at 250 °C. An 80‐nm‐thick Cr mask layer was subsequently deposited by an e‐beam evaporator (EI‐5K, ULVAC) at a rate of 2 Å s^−1^.

Then, using the Langmuir–Blodgett self‐assembly technique, PS (Alfa Aesar Inc.) nanospheres with diameters of 750 nm that were hexagonally close‐packed were transferred from the surface of water onto the substrate by the scooping transfer technique. The size of the PS nanospheres was reduced using O_2_ plasma by RIE (Plasmalab 800 Plus, Oxford Instruments). After the previously deposited Cr layer was etched by ICP‐RIE (Plasma System 100, Oxford Instruments) using Cl_2_ and Ar as etching gases, the SiO_2_ layer was etched by RIE using CF_4_ and CHF_3_ as etching gases to make a patterned hard mask for the GaN etching. PS nanospheres on hard masks were removed during sonication in chloroform. Lastly, the GaN epilayer was dry etched vertically along the patterned mask via ICP‐RIE (TE3100, Tainics) using Cl_2_ and BCl_3_ (30 sccm and 10 sccm, respectively) as etching gases with a source power of 1000 W and a bias power of 200 W. After the dry etching process, residual SiO_2_ hard masks were removed using a buffered oxide etchant (BOE, 6:1, J. T. Baker Inc.). Ag thin film (Ag TF) was deposited onto the substrate using an e‐beam evaporator (EI‐5K, ULVAC) at a rate of 2 Å s^−1^ (Figure S19, Supporting Information).

### Fabrication of Metal Plasmonic Nanostructure on Semiconductor Substrate

#### Sub‐20 nm Master Template Preparation

A Si master template with 25‐nm‐wide lines was fabricated via the directed self‐assembly of BCP. Polystyrene‐b‐polydimethylsiloxane (PS‐b‐PDMS) with a molecular weight (MW) of 48 kg mol^−1^ was purchased from Polymer Source Inc. and used without any purification. A BCP solution of 0.8 wt% was prepared by dissolving BCPs in a mixed solvent of toluene, heptane, and propylene glycol monomethyl ether acetate (PGMEA) (1:1:1 by volume). Si substrates with a width of 1 µm, a depth of 40 nm, and a period of 1.25 µm were prepared by conventional photolithography. The Si substrate surface was treated with a PDMS‐OH brush with a MW of 5 kg mol^−1^ (Polymer Source Inc.) at 150 °C in a vacuum oven. The BCP solution was then spin‐cast on the substrate followed by solvent annealing in a stainless‐steel chamber at room temperature using toluene vapor. The substrate was then etched by an ICP‐RIE with a CF_4_ plasma (etching time = 20 s, gas flow rate = 30 sccm, working pressure = 15 mTorr, and plasma source power = 50 W) followed by O_2_ plasma (etching time = 30 s, gas flow rate = 30 sccm, working pressure = 15 mTorr, and plasma source power = 60 W).

*Fabrication of Plasmonic Ag Nanowires (NWs)* via *S‐nTP*: We fabricated Ag NWs on various substrates by solvent‐assisted nanotransfer printing by following procedures described in our previous work.^[^ ^8^ ^]^ Briefly, the surface of the master template was treated with the PDMS‐OH brush (5 kg mol^−1^, Polymer Source Inc.). PMMA (Sigma‐Aldrich Inc.) with a MW of 100 kg mol^−1^ was dissolved in a mixed solvent of toluene, acetone, and heptane (4.5:4.5:1 by volume) to yield 4 wt% solutions. The solution was spin‐coated onto the master template with a spin speed of 3000 rpm. A PI adhesive tape was evenly attached onto the substrate, and gently removed from the substrate. Ag NWs were formed via the oblique‐angle deposition of Ag onto the polymer film by e‐beam evaporation. The deposition angle was set to 80°, and the deposition rate was kept under 0.2 nm s^−1^. For the printing process, acetone/heptane vapor was applied to the polymer film by putting it in a stainless‐steel chamber saturated with a solvent vapor preheated to 55 °C. After 22 s, the film was gently pressed onto a receiver substrate, and the adhesive tape was gently detached from the receiver substrate. Uniformly distributed Ag NWs were obtained by washing away the polymer film using toluene.

### Samples for Raman Measurements

Sample substrates were divided into three groups: sapphire, GaN epilayer, and GaN NPs. Each group was made up of specimens with three conditions: a pristine sample, a sample with an Ag thin film (Ag TF), and a sample with Ag NWs. A pristine Si sample was prepared separately as a reference substrate to calculate the SEF of each sample. Analyte solutions were prepared for each concentration by dissolving the Rhodamine 6G (R6G) powder into ethanol from 10^−2^–10^−14^ m. Subsequently, 7 µL of R6G solution was drop‐cast on a prepared SERS substrate and dried for 30 m.

Similarly, MNZ (Sigma‐Aldrich Inc.) and TC (Sigma‐Aldrich Inc.) were dissolved in DI water and prepared for each concentration from 10^−2^–10^−10^ m. Then, 7 µL of the solution was drop‐casted on AoG (Ag NWs on GaN NPs) samples and dried for 1 h. For the quantification of MNZ in aqueous solution, Flasinyl tablets (HK inno.N Corp.) consisting of 250 mg of MNZ were dissolved in 100 L of tap water containing soil and sand to simulate environmental antibiotic samples in real rivers. The solution was dried for 1 h, and the SERS spectra of the Flasinyl tablets were acquired on the AoG substrate. For the multiplexing experiment, MNZ and TC solutions of 10^−6^ m were mixed in ratios of 100:0, 75:25, 50:50, 25:75, and 0:100. After 7 µL of the solution was drop‐casted on AoG samples, the substrate was dried for 1 h. The SERS signal of the AoG substrate stored in an ambient condition was maintained for more than 20 days (Figure S17, Supporting Information). With proper washing steps, the AoG substrate was also reusable for various analytes (Figure S18, Supporting Information).

### Characterization

#### Raman Measurement

Using an optical microscope equipped with a 532 nm continuous‐wave laser (excitation power of 120 µW) and a monochromator (Solis 303i, Andor), high‐frequency Raman measurements were performed. The signal was gathered via an objective lens (100 ×, N. A. = 0.9) for 1 s and dispersed via a 1200 line mm^−1^ grating for high‐frequency Raman.

*Calculation of Areal Density of Analyte Molecules*: The area of the SERS substrates was 0.25 cm^2^. A 7 µL drop of analyte (R6G) solution was cast on each sample whose entire surface was then uniformly covered with the solution. After the solution was completely dried, the average areal density of the R6G was calculated using the following equation:

(3) $$\mathrm{Average}\;\mathrm{areal}\;\mathrm{density}\;\mathrm{of}\;\mathrm{the}\;R6G=cN_{A}V/A$$

In the equation, *c*, *N*_A_, *V*, and *A* represent the molar concentration of R6G in the solution, Avogadro's number, the volume of the solution drop, and the surface area of the SERS substrate, respectively.

*Estimation of Substrate Enhancement Factor (SEF)*: To calculate SEF using obtained SERS intensities, we compared SERS signals from nanostructures with ordinary Raman signals from R6G molecules on a bare Si substrate. SEF can be derived via the following equation:

(4) $$\mathrm{SEF}=\left(I_{SERS}/I_{Normal}\right)\times \left(N_{Film}/N_{SERS}\right)$$

In the equation, *I*_SERS_, *I*_Normal_, *N*_Film_, and *N*_SERS_ indicate the intensity of the SERS signal, the intensity of the Raman signal, the number of molecules of the R6G film on the Si wafer, and the number of molecules on the SERS system, respectively. *I*_Normal_ for 10^−2^ m R6G on the Si wafer was 1013.051 counts, and this value was used for all calculations.

*N*_SERS_ is defined as the areal density of the molecules mentioned above, and it was calculated using the following equation:

(5) $$N_{\mathrm{SERS}}=cN_{A}V/A\left(\mathrm{molecules}\;\mu m^{-2}\right)$$

*N*_Film_ was calculated by the following procedure. A high concentration (10^−2^ m) of 7 µL of R6G solution was drop‐casted on the Si substrate of the same size as other SERS substrates, which was followed by complete solvent evaporation for 30 minutes to form a film. N_Film_ corresponds to the areal molecule density of the R6G film and can be estimated as

(6) $$N_{\mathrm{Film}}=hgN_{A}/M\left(\mathrm{molecules}\;\mu m^{-2}\right)$$

In the equation, *h*, *g*, and *M* represent the film height, the density of solid R6G, and the MW (g mol^−1^) of R6G, respectively. Here, we used h = 947.39 nm, g = 1.26 g cm^−3^, and M = 479.02 g mol^−1^. The thickness of R6G was determined from the cross‐sectional SEM image of 10^−2^ m R6G (Figure S11, Supporting Information). The Raman spectra of the R6G on the reference substrate were derived under identical conditions as in the SERS measurements. The SERS intensities of various substrates are provided in the Supporting Information.

*SEM*: SEM images were acquired by a field‐emission SEM (S‐4800, Hitachi) under an acceleration voltage of 10 kV.

*TEM*: Thin lamellae specimens for TEM observation were prepared by a focused ion beam (Helios G4, FEI) using liquid Ga metal as an ion source. Pt was deposited in situ by an equipped gas injection system to protect the sample from structural deformations and milling artifacts. The thickness of Pt was approximately 1 µm. HAADF‐STEM imaging was done by the transmission electron microscope (Talos F200X, FEI) under an acceleration voltage of 200 kV equipped with four Super X SDD EDS detectors. EDS elemental mapping was conducted in the same apparatus with an acquisition time of 600 s.

*Optical Absorption Measurements*: Optical absorption spectra were measured by a UV–vis spectrophotometer (Lambda 1050, Perkin Elmer) under a diffused reflectance mode using an integrating sphere coated with barium sulfate (BaSO_4_).

*XPS*: Valence states of SERS substrates were examined by an X‐ray photoelectron spectrometer (K‐alpha, Thermo VG Scientific). Before the survey scan, surface contamination was cleaned by an Ar cluster gun for 60 s. All spectra were calibrated using the adventitious carbon peak (284.8 eV).

## Conflict of Interest

The authors declare no conflict of interest.

## Supporting information

Supporting Information

## Data Availability

Research data are not shared.
