# Supplementary material for: Synergistic SERS Enhancement in GaN‐Ag Hybrid System toward Label‐Free and Multiplexed Detection of Antibiotics in Aqueous Solutions
Source: Adv Sci (Weinh). 2021 Aug 7;8(19):2100640. doi: 10.1002/advs.202100640 (PMC8498916; doi:10.1002/advs.202100640)
Supplement: Supplementary file 1 — Supporting Information [file ADVS-8-2100640-s001.pdf]

© 2021 Wiley-VCH GmbH

ADVANCED SCIENCE Open Access

Supporting Information

for *Adv. Sci.,* DOI: 10.1002/advs.202100640

Synergistic SERS Enhancement in GaN-Ag Hybrid System toward Label-Free and Multiplexed Detection of Antibiotics in Aqueous Solutions

Kang Hyun Lee, Hanhwi Jang, Yoon Seok Kim, Chul-Ho Lee, Seunghee H. Cho, Minjoon Kim, Hoki Son, Kang Bin Bae, Dung Van Dao, Yeon Sik Jung, and In-Hwan Lee**

Supporting Information

Synergistic SERS Enhancement in GaN-Ag Hybrid System toward Label-Free and Multiplexed Detection of Antibiotics in Aqueous Solutions

Kang Hyun Lee, Hanhwi Jang, Yoon Seok Kim, Chul-Ho Lee,Seunghee H. Cho, Minjoon Kim,Hoki Son, Kang Bin Bae, Dung Van Dao, Yeon Sik Jung,[*] *and In-Hwan Lee*[*]

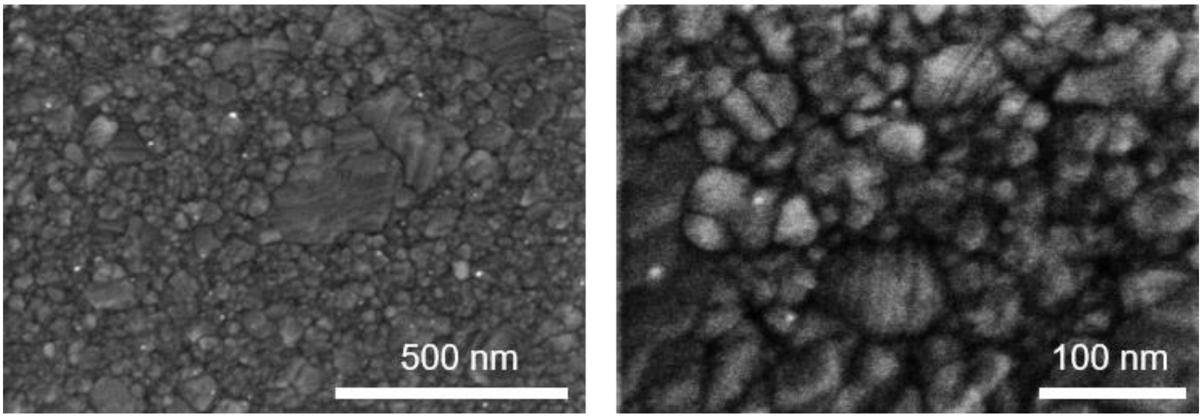

Figure S1. SEM images at low and highmagnification of as-deposited Ag thin film on aGaN epilayer. The high surface roughness of the film may account for increasingly higher SERS performance.

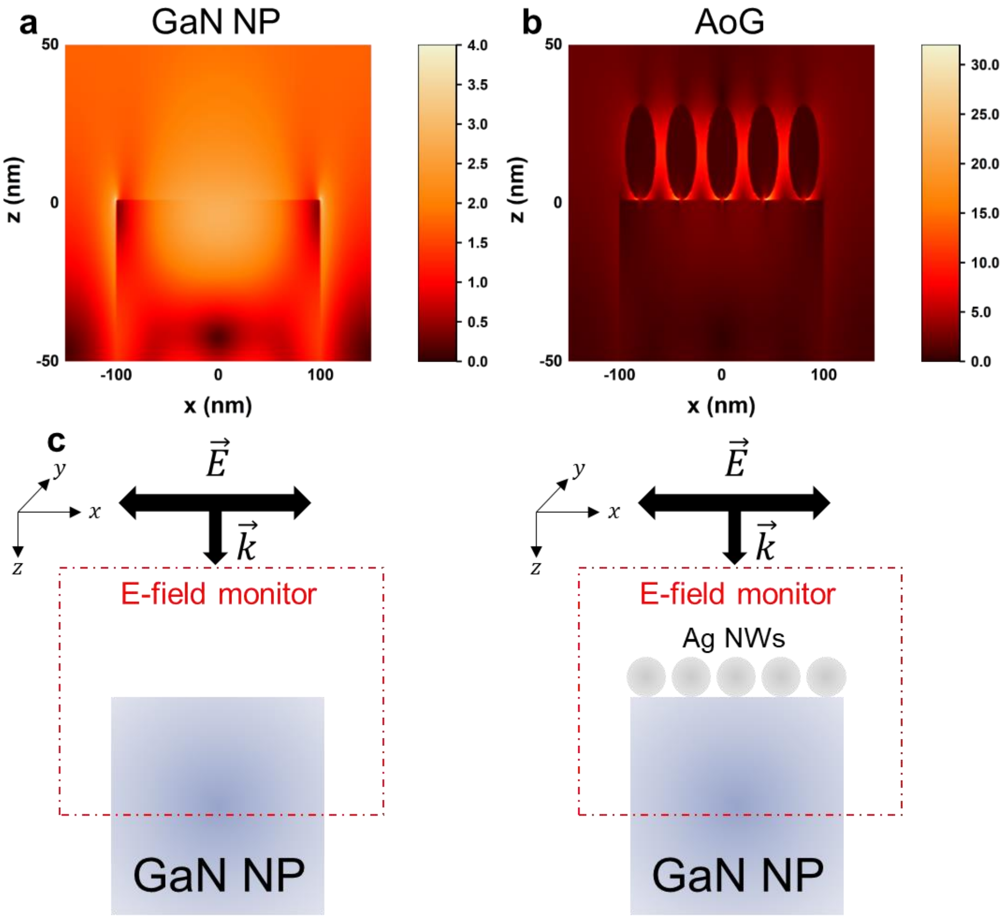

Figure S2. Finite-difference time-domain (FDTD) simulation results of electric field intensity distribution for (a) the GaN nanopillar and (b) the AoG structure under 532 nm laser excitation. The maximum normalized E-field intensity in GaN NP and AoG is 3.98 and 33.2, respectively. (c) Simulation setup for the GaN NP and the AoG structure.

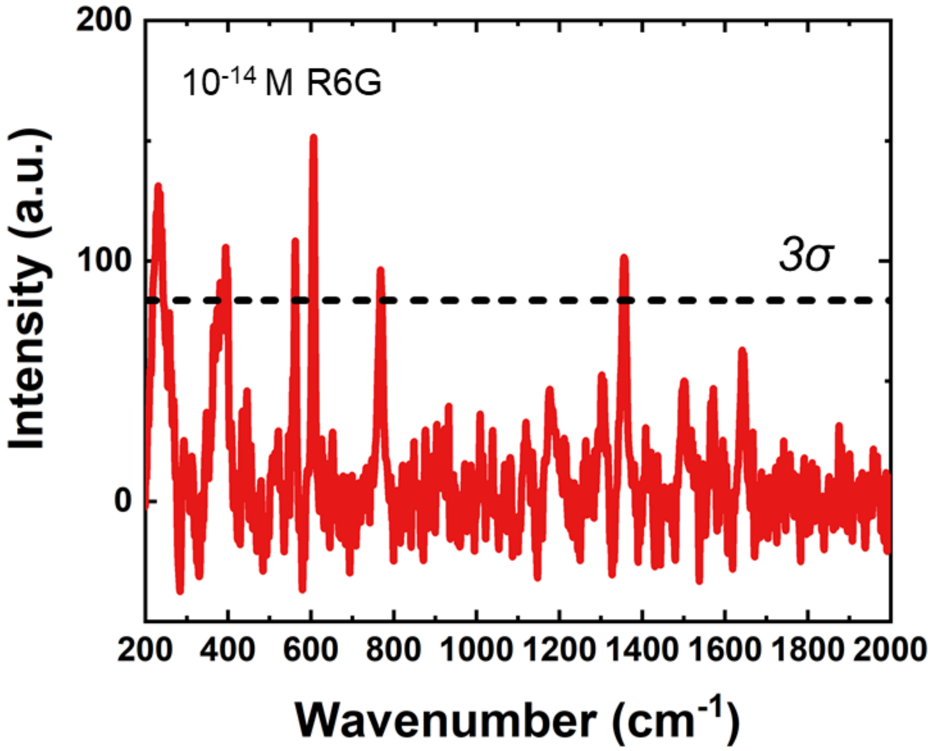

Figure S3. SERS spectrum for 10^{-14} M of R6G adsorbed on the AoG structure. Major peak intensities exceed the magnitude of threesigma.

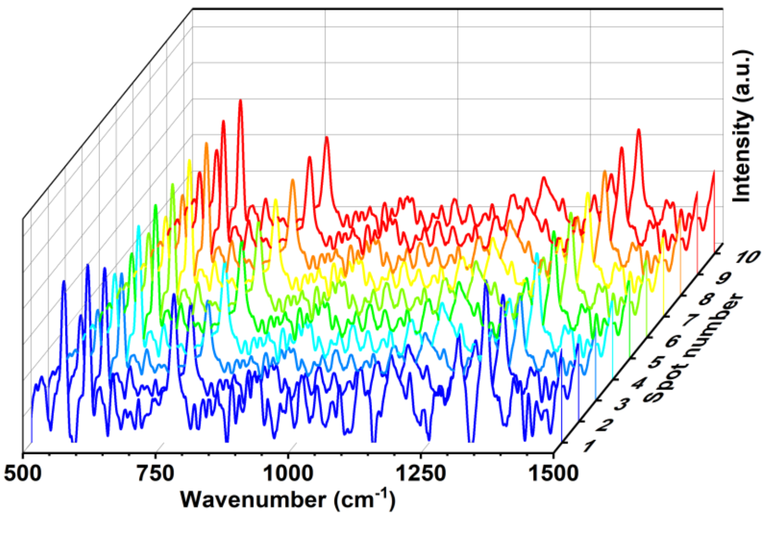

Figure S4. Highly reproducible SERS signals of R6G (10^{-14} M) adsorbed on the AoG structure. Ten spectra were acquired from ten random spots with an integration time of 1 s.

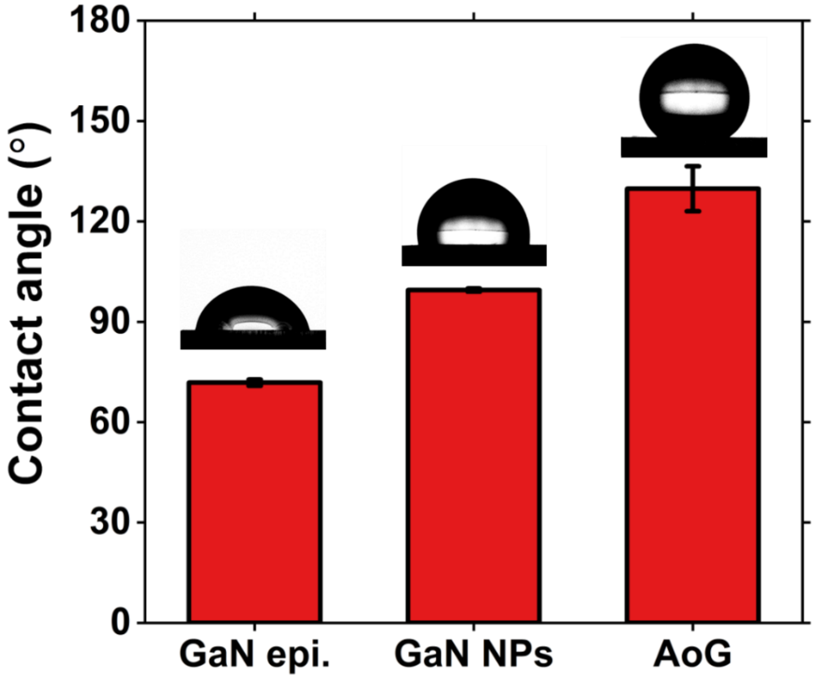

Figure S5. Contact angle measurement of a water droplet on a GaN epilayer, GaN NP, and AoG substrate. Nanostructures can increase the hydrophobicity of the substrate and contribute to concentrating the analyte molecules.

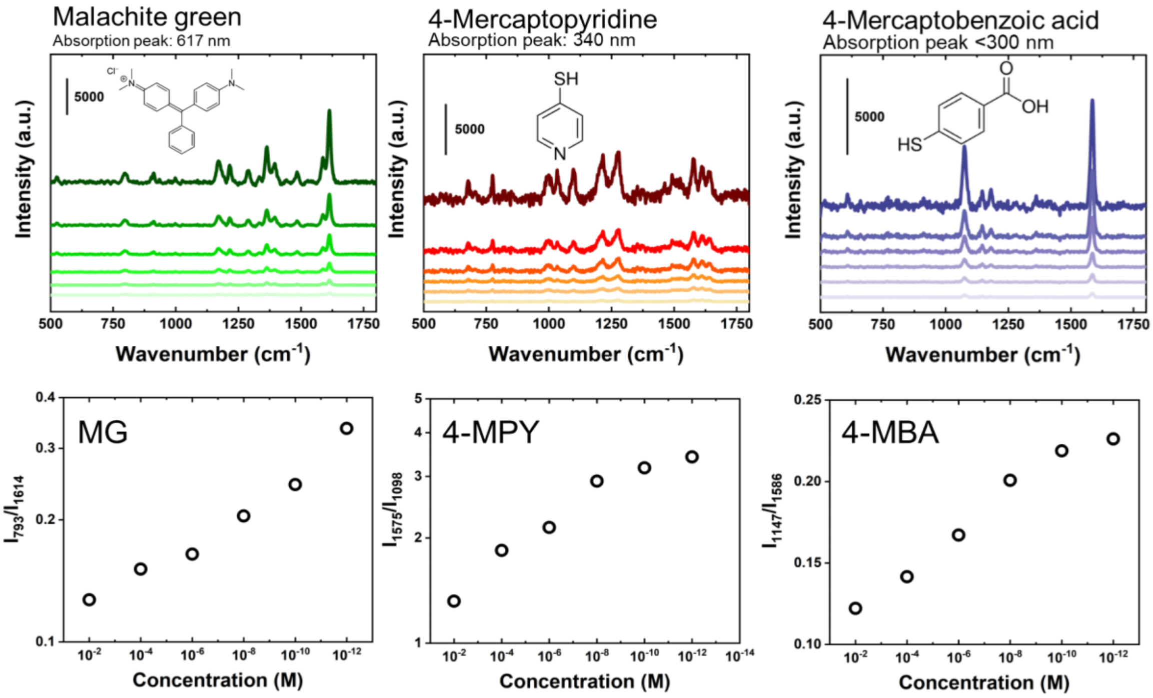

Figure S6. SERS spectra of malachite green, 4-mercaptopyridine, and 4-mercaptobenzoic acid adsorbed on AoG under 532 nm laser excitation. The concentration of each analyte ranges from 10^{-2}–10^{-12} M. The intensity ratio between symmetric and asymmetric vibrations was also plotted for each analyte.

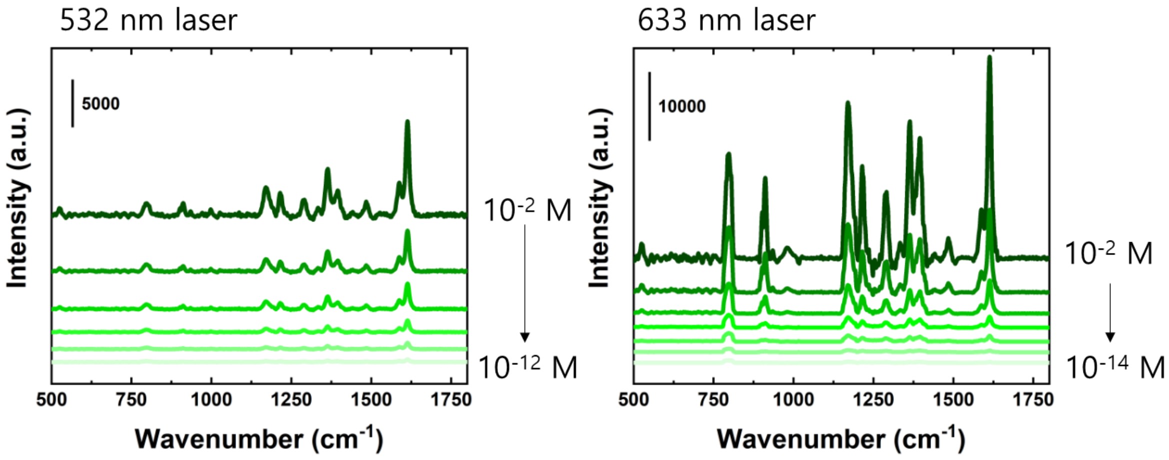

Figure S7. SERS spectra of malachite green adsorbed on AoG under 532 nm and 633 nm laser excitation, showing the increased sensitivity of the AoG substrate under the SERRS condition.

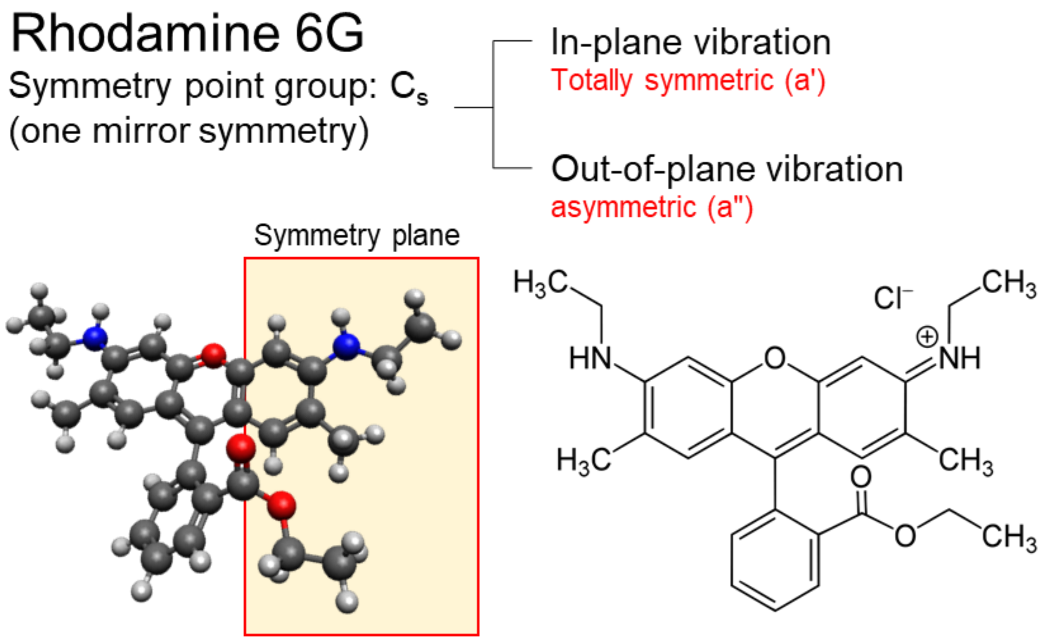

Figure S8. Molecular structure, symmetry, and vibrational characteristic of R6G. Due to Herzberg–Teller selection rule, Raman intensities of out-of-plane vibrations are enhanced by the charge transfer process.

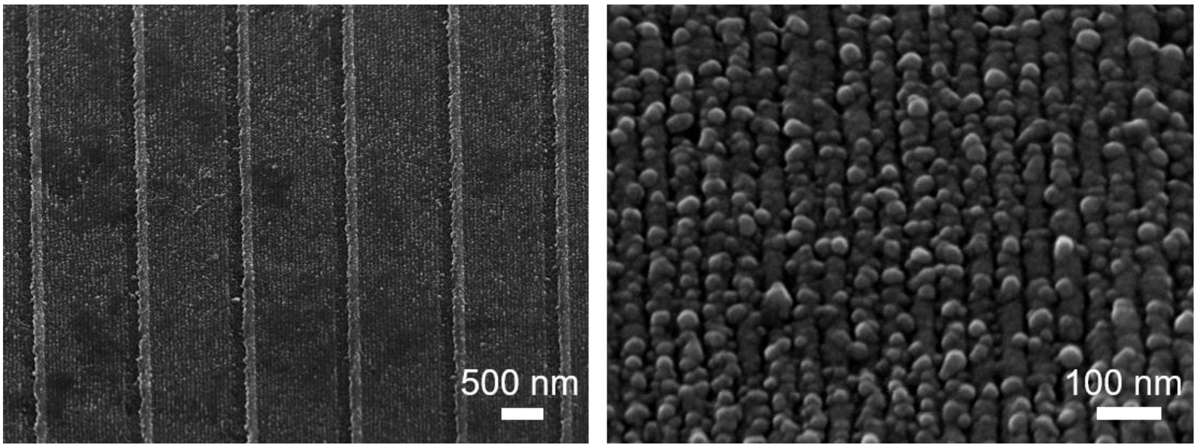

Figure S9. SEM images of printed Ag NWs on GaN epilayer. Compared to AoG, Ag NWs printed on theGaN epilayer are not divided into several Ag nanorods.

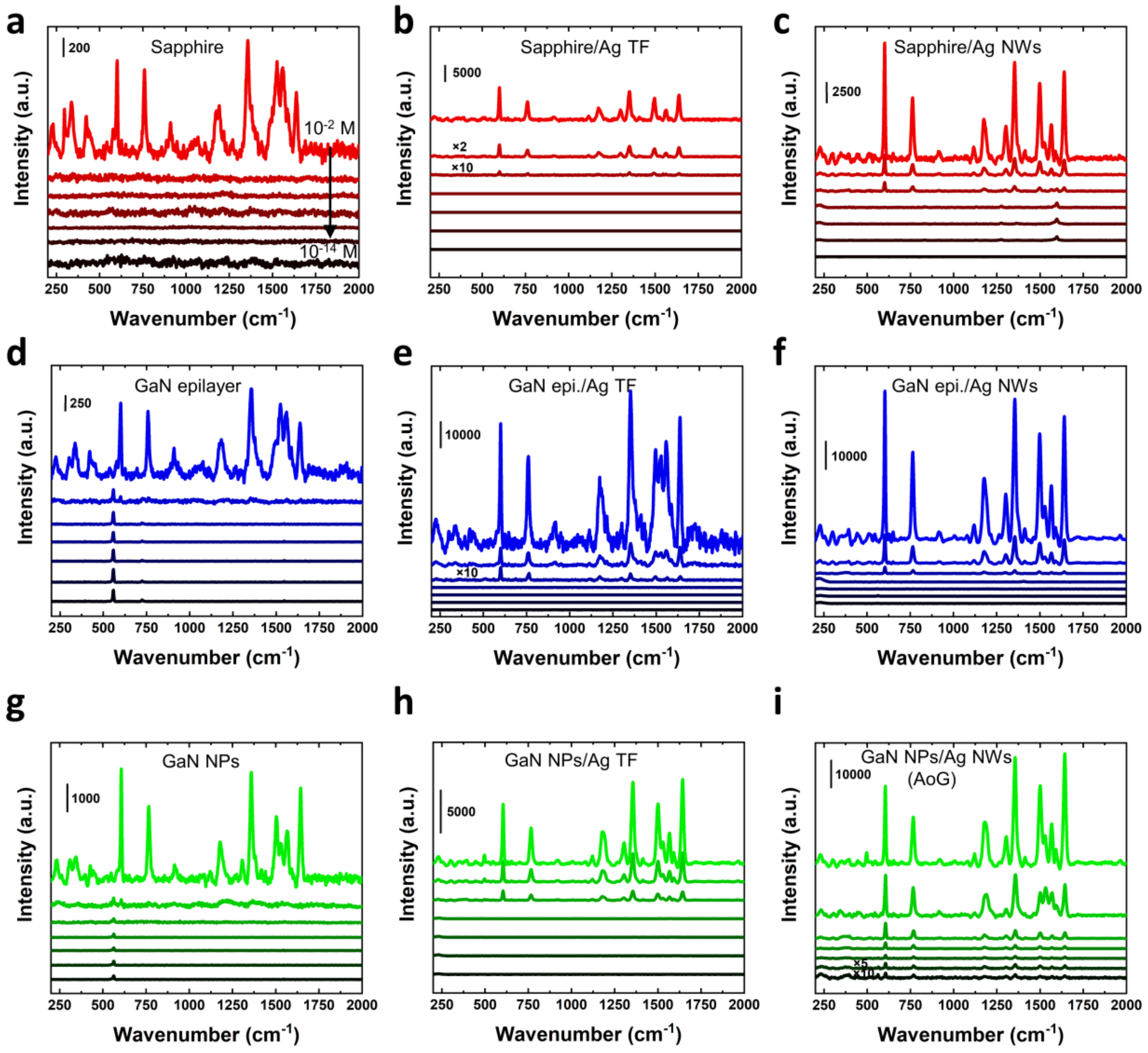

Figure S10. SERS spectra of R6G from 10^{-2}–10^{-14} M for various substrates.

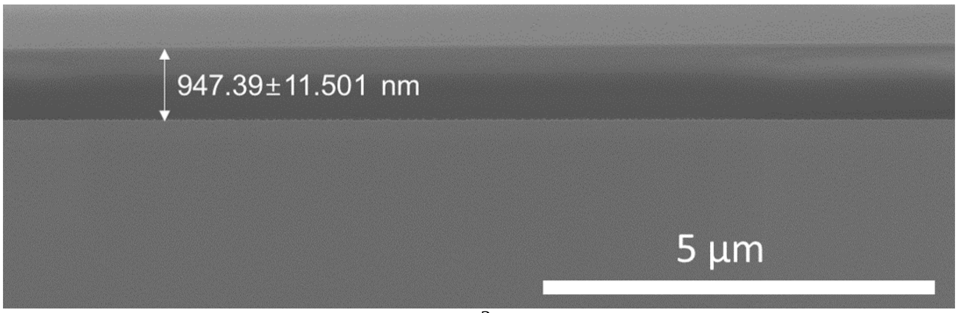

Figure S11. Cross-sectional SEM image of 10^{-2} M R6G. The average film thickness from 30 spots is 947.39 nm, and the standard deviation is 11.501 nm.

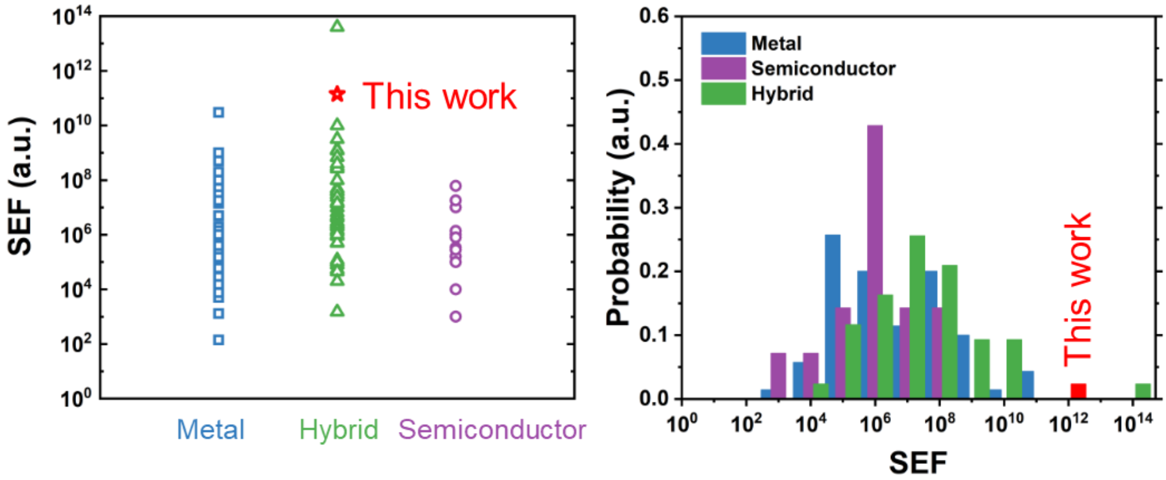

Figure S12. Comparison of SERS SEF of metal-, semiconductor-based and hybrid SERS systems.

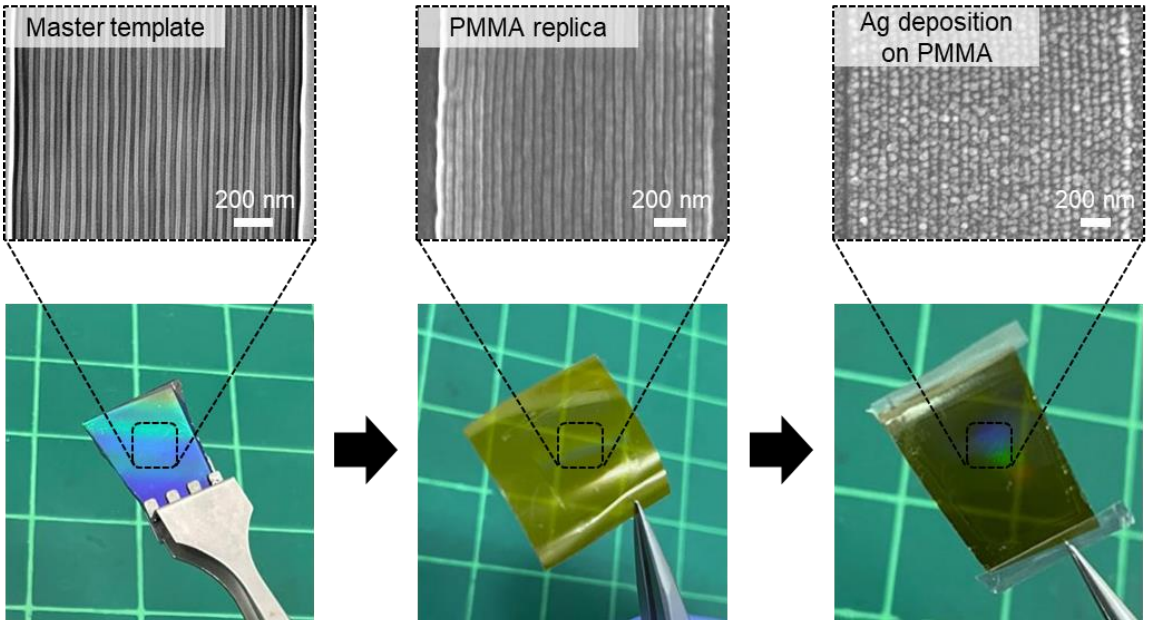

Figure S13. Fabrication procedure for Ag NWs using S-nTP. The 1 μm-wide Si trench was fabricated by conventional photolithography. Then, sub-20-nm SiO_x line patterns were formed via directed self-assembly (DSA) of polystyrene-b-polydimethylsiloxane (PS-b-PDMS). This pattern was replicated by PMMA, and Ag NWs were fabricated on PMMA replicas by following the oblique-angle deposition of Ag.

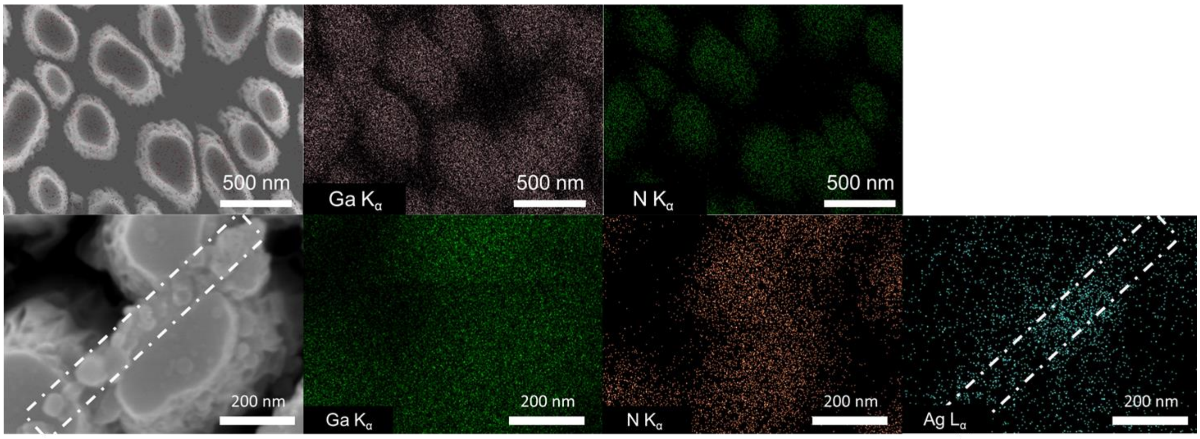

Figure S14. SEM-EDS elemental mapping of GaN NPs and the AoG structure. The white dashed line denotes the region of Ag NW.

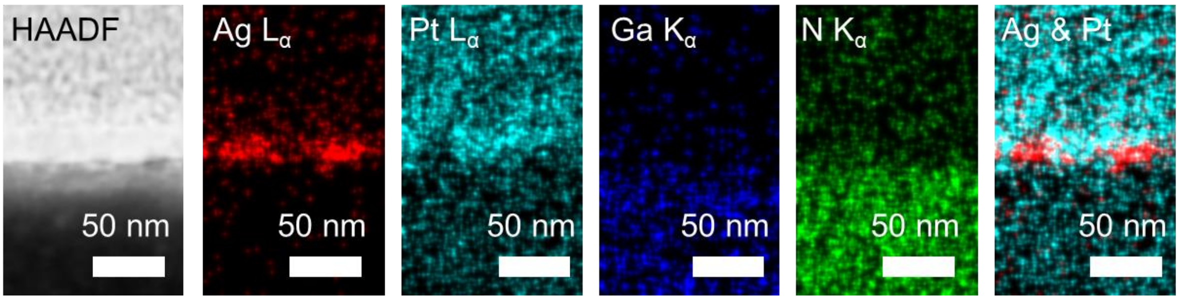

Figure S15. Cross-sectional STEM-EDS elemental mapping of the AoG structure.

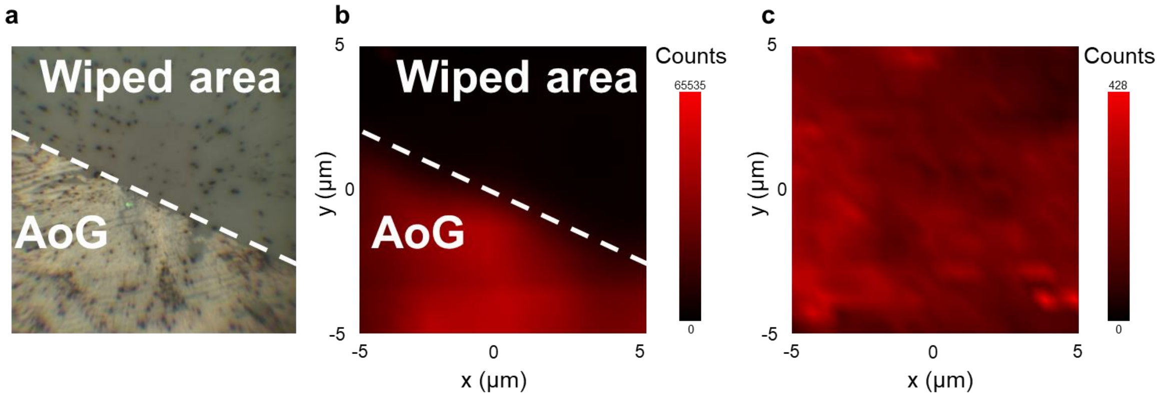

Figure S16. (a)Optical microscope image and Raman mapping of (b) 10^{-4} and (c) 10^{-14} M R6G absorbed on the AoG structure over the 100-μm^2-wide area. The AoG structures in the upper right region of (a) was intentionally removed to confirm the SERS signal amplification effect of Ag NWs on GaN NPs.

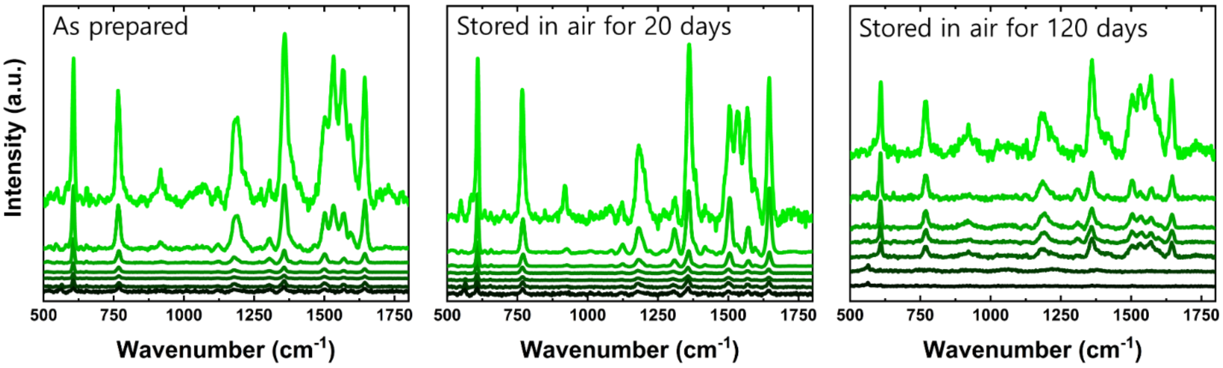

Figure S17. Oxidation stability of the AoG substrate. R6G spectra were acquired from AoG stored in an ambient condition. The R6G concentration ranges from 10^{-2}–10^{-14} M. The signal intensity of 10^{-12} and 10^{-14} M was multiplied by 5 and 10 to improve the visibility of the spectra.

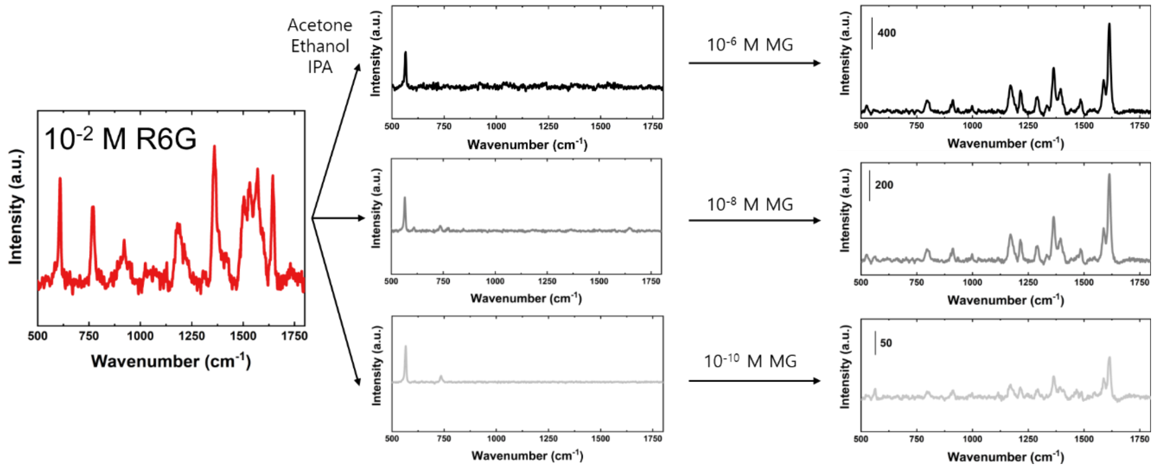

Figure S18. Reusability test conducted on the AoG substrate. After dropping high-concentration (10^{-2} M) R6G, the substrate was cleaned by ultrasonication using acetone, ethanol, and IPA each for 10 minutes. The peaks in cleaned substrates are emerging from GaN and some residual impurities. The characteristic peaks from malachite green can be clearly visible where the malachite green concentration ranges from 10^{-6}–10^{-10} M.

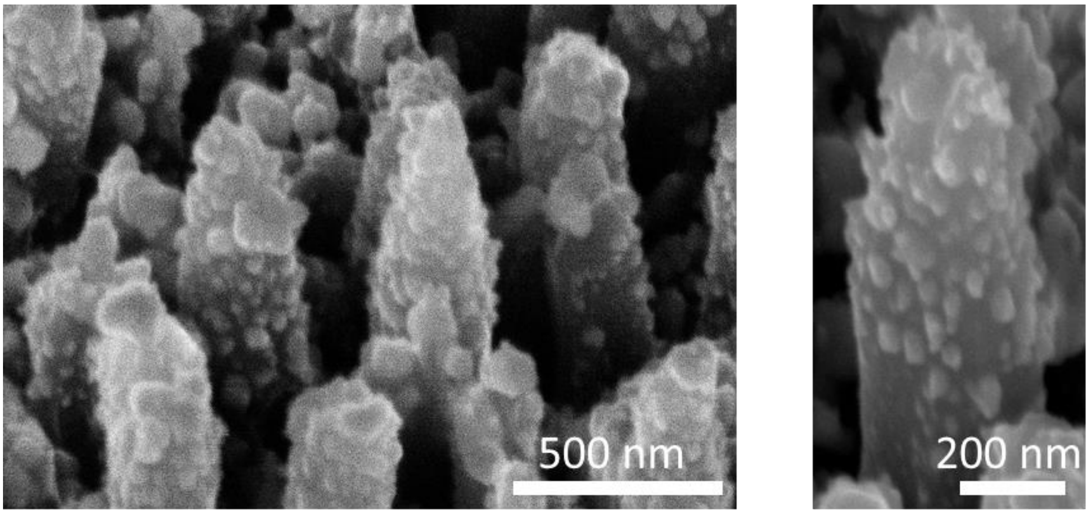

Figure S19. Tilted SEM images of GaN NPs with Ag TF with a tilting angle of 45°.

Table S1. SERS intensities of R6G at a wavenumber of 613 cm^{-1} for various substrates

C **(M)**	**I_{SERS} (counts)**		
	**GaN NP**	**GaN NP+Ag TF**	**GaN NP+Ag NW (AoG)**
**10^{-2}**	4216.76413	25685.0626	30318.3821
**10^{-4}**		8842.00920	13149.5375
**10^{-6}**		3950.83799	5057.82589
**10^{-8}**	No signal		1987.31268
**10^{-10}**		No signal	1022.19983
**10^{-12}**			355.076350
**10^{-14}**			157.282850

Table S2. Characteristic Raman peak positions and corresponding vibrational modes of Rhodamine 6G[1]

Peak position (cm^{-1})	Vibrational mode
613	C-C-C ring out-of-plane bending
772	C-H out-of-plane bending
1127	C-H in-plane bending in xanthene/phenyl rings
1187	C-H in-plane bending in xanthene ring
1312	Hybrid mode (xanthene/phenyl rings and NHC_2H_5 group)
1361	C-C stretching in xanthene ring
1510	
1571	C-C stretching in phenyl ring
1648	C-C stretching in xanthene ring

Table S3. Characteristic Raman peak positions and corresponding vibrational modes of tetracycline[2]

Peak position (cm^{-1})	Vibrational mode
616	Amid-ONH in-plane bending
742	Amin-NH out-of-plane swing
947	C-O stretching
1058	C-Ostretching
1199	C-O stretching
1332	C-C stretching
1444	C-H bending

Table S4. Characteristic Raman peak positions and corresponding vibrational modes of metronidazole[3]

Peak position (cm^{-1})	Vibrational mode
841	C-H rocking
996	C-H wagging and O-H bending
1141	O-H bending and C-H twisting
1283	O-Hbending, C-H in-plane bending, and C-H twisting
1372	C-H stretching and C-H in-plane bending

Table S5.Substrateenhancementfactorsformetal-basedSERSsubstrates

Ref.	System	Analyte	λe·c/nm	SEF
[4]	AuNRs	Cy3	633	$1.4 \cdot 10^{2}$
[5]	AuNRs	ATP	785	$5.2 \cdot 10^{2}$
[6]	Optically aggregated AuNRs	BSA	633	10^{3}-10^{4}
[4]	Undulated AuNRs@Au	Cy3	633	$1.6 \cdot 10^{4}$
[7]	AuNstars	MBA	633	$2\text{-}5 \cdot 10^{4}$
[5]	Au bipyramids	ATP	785	$3.0 \cdot 10^{5}$
[8]	Ag@Au nanoplates	CV	785	$0.5 \cdot 10^{5}$
[8]	Ag nanoplates	CV	785	$0.2 \cdot 10^{5}$
[9]	Au cuboids	2NAP	785	$0.1\text{-}1.2 \cdot 10^{9}$
[10]	Ag cuboctahedra	BDT	532	$5.0 \cdot 10^{4}$
[10]	Ag cuboctahedra	BDT	785	$6.5 \cdot 10^{4}$
[10]	Ag@Au cuboctahedra	BDT	532	$4.4 \cdot 10^{4}$
[10]	Ag@Au cuboctahedra	BDT	785	$4.8 \cdot 10^{6}$
[11]	AuNSs	ATP	647	$2.7 \cdot 10^{4}$
[11]	AuNSs	NTP	647	$1.3 \cdot 10^{4}$
[12]	AuNSs	ATP	785	$1 \cdot 10^{4}$
[13]	AuNS (single-particle)	TAMRA	532	$7.5 \cdot 10^{3}$
[13]	AuNS self-similar nanolenses (single-particle)	TAMRA	532	$1.4 \cdot 10^{6}$
[14]	AuNS@AuNSs superstructures	MBA	785	$1.4 \cdot 10^{7}$
[15]	AuNS@AuNSs superstructure (single-particle)	MBA	633	$0.9\text{-}2.1 \cdot 10^{5}$
[16]	PtAuNS bridged hexamers	2NAP	785	$1.9\text{-}8.4 \cdot 10^{6}$
[14]	AuNRs	MBA	785	$1.9 \cdot 10^{6}$
[14]	AuNR@AuNSs superstructures	MBA	785	$3.5 \cdot 10^{7}$
[15]	AuNstar (single-particle)	MBA	633	$2.5\text{-}3.5 \cdot 10^{4}$

[12]	AuNstars	MBA	785	$1\cdot10^{8}$
[12]	AuNstar@AuNSs superstructures	ATP/MBA	785	$0.5\text{-}5\cdot10^{10}$
[17]	AuNTs nanoplates	CV	633	$3.8\cdot10^{4}$
[17]	AuNTs@Au nanoplates	NTP	514, 633	$1.4\cdot10^{6}$
[17]	AuNTs@Au nanoplates	CV	514, 633	$1.2\text{-}2.3\cdot10^{5}$
[18]	Undulated AuNTs@Ag nanoplates	R6G	532	$3.5\text{-}5.1\cdot10^{5}$
[19]	AuNS@Au/Ag nanostars	CV, MG	633	10^{9}
[20]	Au@Ag cuboids	MBA	532	$7.8\cdot10^{4}$
[21]	AgNcube@AuNSs superstructures (single-particle/AFM)	R6G	532	$2\cdot10^{8}$
[21]	AgNcube@AuNSs superstructures	ATP	532	10^{6}
[22]	AuNSs pen-on-paper multilayers	MG	532	$2\cdot10^{5}$
[22]	AuNSs pen-on-paper multilayers	MG	785	$1.5\cdot10^{5}$
[23]	AuNS / AgNS close-packed on substrate	R6G	633	10^{6}
[24]	AgNstars close-packed monolayer	Phos, Carb	633, 785	$1.7\cdot10^{7}$
[25]	AuNstars on Au mirror	MBA	785	10^{9}
[26]	Asymmetric AuNstars on Au mirror	MBA	785	$0.2\text{-}2.9\cdot10^{8}$
[26]	Symmetric AuNstars on Au mirror	MBA	785	$2.3\text{-}7.5\cdot10^{8}$
[27]	AuNTs close-packed monolayer	NTP	785	$2.8\cdot10^{4}$
[28]	AuNTs close-packed monolayer	NAP	633	$0.8\text{-}2.0\cdot10^{3}$
[28]	AuNTs close-packed monolayer on Au mirror	NAP	633	$1.1\text{-}2.1\cdot10^{4}$
[29]	Undulated AuNTs@Au close-packed multilayer	NTP	785	$6\cdot10^{4}$
[30]	Undulated AuNTs@Au close-packed monolayer	NTP	785	$5.6\cdot10^{5}$
[31]	Au@Au cuboids close-packed monolayer	R6G	785	$5.0\cdot10^{7}$
[31]	Au@Ag dumbbells close-packed monolayer	R6G	785	$2.9\cdot10^{7}$
[32]	Ag layer on stainless steel wire mesh	MBA	785	$10^{3}\text{-}10^{6}$
[33]	Au@Ag cuboid close-packed monolayer	MBA	785	$3.9\cdot10^{5}$
[34]	Self-similar Ag nanolens substrate	R6G	514	$1.8\cdot10^{7}$
[35]	Ag nanocrystalline bulk	R6G	514	$1.4\cdot10^{10}$
[36]	Ag dendrites on Cu grids	R6G	785	$6.1\cdot10^{5}$
[37]	Ag nanoparticles	R6G	785	$1.5\cdot10^{7}$
[38]	Rough Au film	R6G	633	$7.07\cdot10^{9}$
[39]	Confeito-like Au NPs	R6G	633	10^{5}
[40]	Au 3D cross-point nanonetwork	R6G	633	$4.1\cdot10^{7}$
[41]	Ag nanoneedles	R6G	532	$1.2\cdot10^{10}$
[42]	AgNPs	R6G	532	$3.65\cdot10^{8}$
[43]	Ag nanodishes	R6G	532	$6.17\cdot10^{7}$
[38]	Superwettable Au dendrites	R6G	532	10^{8}

[44]	Ag/Au bimetallic nanoalloys	R6G	532	$9\cdot10^4$

Table S6.Substrateenhancementfactorsforsemiconductor-basedSERSsubstrates

Ref.	System	Analyte	λe·c/nm	SEF
[45]	amorphous Rh_3S microbowls	4NBT, R6G	514, 633, 647, 785	10^5
[46]	$AgFeO_2$ nanoparticles	R6G, 4-MBA	633	10^7
[47]	Oxygen incorporated MoS_2	R6G	532	$1.6\cdot10^5$
[48]	Twin nanowires ZnSe	R6G, CV	532	$6.12\cdot10^7$
[49]	Nanowire $W_{18}O_{49}$	R6G	532	$3.40\cdot10^5$
[50]	porous carbon nanowire	R6G, β-lactoglobulin, glucose	532, 785	$1.00\cdot10^6$
[51]	MoO_3 quantum dots	R6G	532	$1.00\cdot10^6$
[52]	α-MoO_{3-x} nanobelt	R6G, 4-MBA, MB	532, 633, 785	$1.8\cdot10^7$
[53]	3D ZnO nanodendrite	R6G	1030	$1.37\cdot10^6$
[54]	Superstructure Cu_2O	R6G, CV	647	$8.0\cdot10^5$
[55]	Concave sphere Cu_2O	R6G, CV	514.5, 647	$2.8\cdot10^5$
[56]	20 nm nanocrystal ZnO	4-MPY/BVPP	514.5	$1.0\cdot10^3$
[57]	Sphere/spindle/cube Fe_2O_3	4-MPY	514.5	$1.0\cdot10^4$
[58]	300 nm nanospheres Cu_2O	4-MBA	488	$1.0\cdot10^5$

Table S7.Substrateenhancementfactorsfor hybridSERSsubstrates

Ref.	System	Analyte	λe·c/nm	SEF
[59]	AuNS@pNIPAM core/shell microgels	1NAT	785	$5.2\cdot10^5$
[60]	AuNSs@silica	MBA	785	$4.5\cdot10^4$
[5]	AuNR tip-to-tip linked oligomers	ATP	785	$1.0\cdot10^6$
[61]	AuNstars on PS microbeads	MBA	785	$0.7\text{-}7.5\cdot10^7$
[5]	Au bipyramid tip-to-tip linked oligomers	ATP	785	$1.0\cdot10^7$
[62]	AuNS@organosilica	R6G	633	$5.1\cdot10^5$
[62]	AuNS@organosilica dimer	R6G	633	$6.6\cdot10^6$
[62]	AuNS@organosilica trimer	R6G	633	$1.6\cdot10^7$
[63]	AuNSs@silica janus as dimers	R6G	532	$4.9\cdot10^6$
[63]	AuNSs@silica janus as dimers	R6G	633	$2.6\cdot10^6$
[63]	AuNSs@silica janus as dimers	R6G	785	$4.2\cdot10^6$
[64]	AgNPs on nano-rippled substrate	MBN	514	$1.5\cdot10^3$
[64]	AgNPs on nano-rippled substrate	MBN	647	$2\cdot10^4$
[65]	AuNS@AgNSs on cellulose	R6G	785	$4\cdot10^8$
[66]	Au bipyramid close-packed monolayers on ITO	ATP	633	$0.1\text{-}6\cdot10^6$
[66]	Au bipyramid close-packed monolayers on ITO	ATP	782	$0.1\text{-}3.5\cdot10^6$

[66]	Au bipyramid close-packed monolayers on ITO	ATP	830	$0.1\text{-}3.3 \cdot 10^6$
[67]	Nanoweb of SiNSs and AuNSs on substrate	CV	785	$2.2\text{-}3.0 \cdot 10^7$
[67]	Nanoweb of SiNSs and AuNSs on substrate	R6G	785	$1.4 \cdot 10^7$
[68]	AuNS@MoS2 nanoflowers (spray-coated)	R6G	785	10^6
[69]	AuNS@Ag NR capsules adsorbed onto cellulose	2NAT	785	$4 \cdot 10^{13}$
[70]	Ag/ZnO substrate	ATP/PhRed	514	$3.2 \cdot 10^9$
[71]	AgNPs on Ge substrate	R6G	514, 633	$1.3 \cdot 10^9$
[71]	AgNPs on Si substrate	R6G	514, 633	$2.9 \cdot 10^7$
[72]	Nanoporous Au/SnO/Ag film	R6G	532	$1.00 \cdot 10^{10}$
[73]	Au@Ag/3D-Si substrate	R6G	532	$1.20 \cdot 10^9$
[74]	AgNPs/graphene o·ide sheet	R6G, CV	532	$9.00 \cdot 10^5$
[75]	Fe3O4@SiO2@Ag composite microspheres	R6G	514	$1.28 \cdot 10^6$
[76]	graphene/bilayer silver/Cu sandwich structure	R6G, CV	785	$1.19 \cdot 10^5$
[76]	AgD/AgNS/Cu sandwich structure	R6G, CV	785	$8.04 \cdot 10^4$
[76]	AgNS/Cu substrate	R6G, CV	785	$5.37 \cdot 10^4$
[77]	Ag nanoislands on silica spheres	R6G	532	$3.76 \cdot 10^7$
[78]	Ag@InConeNanopore substrate	R6G	532	$2.34 \cdot 10^6$
[79]	3D AgNPs/multilayer graphene o·ide	R6G	532	7×10^8
[79]	3D AgNPs/multilayer graphene o·ide	CV	532	3.2×10^8
[80]	RGO–AgNP	R6G	514	$2.3 \cdot 10^6$
[81]	AgNPs/wheatear-like H-ZnO	R6G	532	$4.9 \cdot 10^7$
[82]	AgNPs/urchin-like ZnO-HNS	R6G	532	10^8
[83]	Ag@ZnO worm	R6G	514	3.027×10^7
[84]	TiO·-NRs/Au-NPs	R6G	532	2.64×10^8
[85]	Ag-coated porous Si photonic crystals	R6G	1064	10^5
[85]	Ag-coated porous Si photonic crystals	CV	1064	$5 \cdot 10^5$

Computational details on finite-difference time-domain (FDTD) simulation

The 3D FDTD simulations were performed by Lumerical FDTD software for two structures: a GaN nanopillar and the AoG structure. The periodicity of Ag nanowires was imposed by applying the periodic boundary conditions on the x- and y-axes, and perfectly matched layers were used on the z-axis boundary condition. Five Ag nanowires were placed

on GaN nanopillar in the AoG structure. The total-field scattered-field source with an E-field amplitude of one was illuminated from the air toward the substrate. The E-field enhancement was obtained by E-field monitor in the x-z plane.

Reference

[1] H. Watanabe, N. Hayazawa, Y. Inouye, S. Kawata, *J. Phys. Chem. B***2005**, *109*, 5012.
[2] D. Jin, Y. Bai, H. Chen, S. Liu, N. Chen, J. Huang, S. Huang, Z. Chen, *Anal. Methods***2015**, *7*, 1307.
[3] C. Han, J. Chen, X. Wu, Y.-w. Huang, Y. Zhao, *Talanta***2014**, *128*, 293.
[4] Y. Shang, J. Shi, H. Liu, X. Liu, Z.-G. Wang, B. Ding, *Nanoscale***2018**, 10, 9455.
[5] R. Pardehkhorram, S. Bonaccorsi, H. Zhu, V. R. Gonçales, Y. Wu, J. Liu, N. A. Lee, R. D. Tilley, J. J. Gooding, *Chemical Communications***2019**, 55, 7707.
[6] B. Fazio, C. D'Andrea, A. Foti, E. Messina, A. Irrera, M. G. Donato, V. Villari, N. Micali, O. M. Maragò, P. G. Gucciardi, *Scientific Reports***2016**, 6, 26952.
[7] C. G. Khoury, T. Vo-Dinh, *The Journal of Physical Chemistry C***2008**, 112, 18849.
[8] S. K. Krishnan, R. Esparza, F. J. Flores-Ruiz, E. Padilla-Ortega, G. Luna-Bárcenas, I. C. Sanchez, U. Pal, *ACS Omega***2018**, 3, 12600.
[9] K.-K. Liu, S. Tadepalli, L. Tian, S. Singamaneni, *Chemistry of Materials***2015**, 27, 5261.
[10] J. Zhang, S. A. Winget, Y. Wu, D. Su, X. Sun, Z.-X. Xie, D. Qin, *ACS Nano***2016**, 10, 2607.
[11] S. Hong, X. Li, *Journal of Nanomaterials***2013**, 2013, 790323.
[12] A. S. D. S. Indrasekara, R. Thomas, L. Fabris, *Physical Chemistry Chemical Physics***2015**, 17, 21133.
[13] C. Heck, J. Prinz, A. Dathe, V. Merk, O. Stranik, W. Fritzsche, J. Kneipp, I. Bald, *ACS Photonics***2017**, 4, 1123.
[14] L. Tian, S. Tadepalli, M. Fei, J. J. Morrissey, E. D. Kharasch, S. Singamaneni, *Chemistry of Materials***2015**, 27, 5678.
[15] V. Tran, C. Thiel, J. T. Svejda, M. Jalali, B. Walkenfort, D. Erni, S. Schlücker, *Nanoscale***2018**, 10, 21721.
[16] S. Yoo, J. Kim, J.-M. Kim, J. Son, S. Lee, H. Hilal, M. Haddadnezhad, J.-M. Nam, S. Park, *Journal of the American Chemical Society***2020**, 142, 15412.
[17] G. Wang, Y. Liu, C. Gao, L. Guo, M. Chi, K. Ijiro, M. Maeda, Y. Yin, *Chem***2017**, 3, 678.
[18] F. Liebig, R. M. Sarhan, C. Prietzel, C. N. Z. Schmitt, M. Bargheer, J. Koetz, *ACS Applied Nano Materials***2018**, 1, 1995.
[19] D. Chen, Z. Song, F. Chen, J. Huang, J. Wei, Y. Zhao, *RSC Advances***2016**, 6, 66056.
[20] S.-R. Wu, X.-D. Tian, S.-Y. Liu, Y. Zhang, J.-F. Li, *Journal of Raman Spectroscopy***2018**, 49, 659.
[21] Z. Huang, G. Meng, Q. Huang, B. Chen, Y. Lu, Z. Wang, X. Zhu, K. Sun, *Journal of Raman Spectroscopy***2017**, 48, 217.
[22] L. Polavarapu, A. L. Porta, S. M. Novikov, M. Coronado-Puchau, L. M. Liz-Marzán, *Small***2014**, 10, 3065.
[23] J. Y. Kim, H. Kim, B. H. Kim, T. Chang, J. Lim, H. M. Jin, J. H. Mun, Y. J. Choi, K. Chung, J. Shin, S. Fan, S. O. Kim, *Nature Communications***2016**, 7, 12911.
[24] J. Sitjar, J.-D. Liao, H. Lee, L. P. Pan, B. H. Liu, W.-e. Fu, G. D. Chen, *Nanomaterials***2019**, 9, 1750.
[25] A. S. D. S. Indrasekara, S. Meyers, S. Shubeita, L. C. Feldman, T. Gustafsson, L. Fabris, *Nanoscale***2014**, 6, 8891.

[26] W. Niu, Y. A. A. Chua, W. Zhang, H. Huang, X. Lu, *Journal of the American Chemical Society* **2015**, 137, 10460.

[27] a) F. Liebig, R. M. Sarhan, C. Prietzel, A. Reinecke, J. Koetz, *RSC Advances* **2016**, 6, 33561; b) F. Liebig, R. M. Sarhan, M. Sander, W. Koopman, R. Schuetz, M. Bargheer, J. Koetz, *ACS Applied Materials & Interfaces* **2017**, 9, 20247.

[28] Y. H. Lee, C. K. Lee, B. Tan, J. M. Rui Tan, I. Y. Phang, X. Y. Ling, *Nanoscale* **2013**, 5, 6404.

[29] F. Liebig, R. M. Sarhan, C. Prietzel, A. F. Thünemann, M. Bargheer, J. Koetz, *Langmuir* **2018**, 34, 4584.

[30] F. Liebig, R. M. Sarhan, C. N. Z. Schmitt, A. F. Thünemann, C. Prietzel, M. Bargheer, J. Koetz, *ChemPlusChem* **2020**, 85, 519.

[31] B. N. Khlebtsov, Z. Liu, J. Ye, N. G. Khlebtsov, *Journal of Quantitative Spectroscopy and Radiative Transfer* **2015**, 167, 64.

[32] T. Szymborski, E. Witkowska, K. Niciński, Z. Majka, T. Krehlik, T. Deskur, K. Winkler, A. Kamińska, *Nanomaterials* **2018**, 8, 663.

[33] X. Yang, J. Li, Y. Zhao, J. Yang, L. Zhou, Z. Dai, X. Guo, S. Mu, Q. Liu, C. Jiang, M. Sun, J. Wang, W. Liang, *Nanoscale* **2018**, 10, 142.

[34] G. Das, S. Alrasheed, M. L. Coluccio, F. Gentile, A. Nicastri, P. Candeloro, G. Cuda, G. Perozziello, E. Di Fabrizio, *RSC Advances* **2016**, 6, 107916.

[35] Z. Yi, Y. Yi, J. Luo, X. Ye, P. Wu, X. Ji, X. Jiang, Y. Yi, Y. Tang, *RSC Advances* **2015**, 5, 1718.

[36] H. S. S. Sharma, E. Carmichael, D. McCall, *Vibrational Spectroscopy* **2016**, 83, 159.

[37] W.-L. Zhai, D.-W. Li, L.-L. Qu, J. S. Fossey, Y.-T. Long, *Nanoscale* **2012**, 4, 137.

[38] J. Wang, C. Qiu, X. Mu, H. Pang, X. Chen, D. Liu, *Talanta* **2020**, 210, 120631.

[39] M. Ujihara, N. M. Dang, T. Imae, *Sensors* **2017**, 17, 2563.

[40] J. W. Jeong, M. M. P. Arnob, K.-M. Baek, S. Y. Lee, W.-C. Shih, Y. S. Jung, *Advanced Materials* **2016**, 28, 8695.

[41] Y. Yang, Z.-Y. Li, K. Yamaguchi, M. Tanemura, Z. Huang, D. Jiang, Y. Chen, F. Zhou, M. Nogami, *Nanoscale* **2012**, 4, 2663.

[42] J. Jiang, S. Wang, H. Wu, J. Zhang, H. Li, J. Jia, X. Wang, J. Liao, *RSC Advances* **2015**, 5, 105820.

[43] W. Tao, A. Zhao, H. Sun, Z. Gan, M. Zhang, D. Li, H. Guo, *RSC Advances* **2014**, 4, 3487.

[44] A. V. Girão, P. C. Pinheiro, M. Ferro, T. Trindade, *RSC Advances* **2017**, 7, 15944.

[45] A. Li, J. Lin, Z. Huang, X. Wang, L. Guo, *iScience* **2018**, 10, 1.

[46] Z. Shi, T. Wang, H. Lin, X. Wang, J. Ding, M. Shao, *Nanoscale* **2013**, 5, 10029.

[47] Z. Zheng, S. Cong, W. Gong, J. Xuan, G. Li, W. Lu, F. Geng, Z. Zhao, *Nature Communications* **2017**, 8, 1993.

[48] G. Wang, H. Wei, Y. Tian, M. Wu, Q. Sun, Z. Peng, L. Sun, M. Liu, *Opt. Express* **2020**, 28, 18843.

[49] S. Cong, Y. Yuan, Z. Chen, J. Hou, M. Yang, Y. Su, Y. Zhang, L. Li, Q. Li, F. Geng, Z. Zhao, *Nature Communications* **2015**, 6, 7800.

[50] N. Chen, T.-H. Xiao, Z. Luo, Y. Kitahama, K. Hiramatsu, N. Kishimoto, T. Itoh, Z. Cheng, K. Goda, *Nature Communications* **2020**, 11, 4772.

[51] J. Zhang, Y. Pan, Y. Chen, H. Lu, *Journal of Materials Chemistry C* **2018**, 6, 2216.

[52] H. Wu, H. Wang, G. Li, *Analyst* **2017**, 142, 326.

[53] R. Haldavnekar, K. Venkatakrishnan, B. Tan, *Nature Communications* **2018**, 9, 3065.

[54] J. Lin, Y. Shang, X. Li, J. Yu, X. Wang, L. Guo, *Advanced Materials* **2017**, 29, 1604797.

[55] X. Li, Y. Shang, J. Lin, A. Li, X. Wang, B. Li, L. Guo, *Advanced Functional Materials* **2018**, 28, 1801868.
[56] Y. Wang, W. Ruan, J. Zhang, B. Yang, W. Xu, B. Zhao, J. R. Lombardi, *Journal of Raman Spectroscopy* **2009**, 40, 1072.
[57] X. Fu, F. Bei, X. Wang, X. Yang, L. Lu, *Journal of Raman Spectroscopy* **2009**, 40, 1290.
[58] L. Jiang, T. You, P. Yin, Y. Shang, D. Zhang, L. Guo, S. Yang, *Nanoscale* **2013**, 5, 2784.
[59] R. A. Álvarez-Puebla, R. Contreras-Cáceres, I. Pastoriza-Santos, J. Pérez-Juste, L. M. Liz-Marzán, *Angewandte Chemie International Edition* **2009**, 48, 138.
[60] B. Mir-Simon, I. Reche-Perez, L. Guerrini, N. Pazos-Perez, R. A. Alvarez-Puebla, *Chemistry of Materials* **2015**, 27, 950.
[61] A. B. Serrano-Montes, J. Langer, M. Henriksen-Lacey, D. Jimenez de Aberasturi, D. M. Solís, J. M. Taboada, F. Obelleiro, K. Sentosun, S. Bals, A. Bekdemir, F. Stellacci, L. M. Liz-Marzán, *The Journal of Physical Chemistry C* **2016**, 120, 20860.
[62] H. Hu, F. Ji, Y. Xu, J. Yu, Q. Liu, L. Chen, Q. Chen, P. Wen, Y. Lifshitz, Y. Wang, Q. Zhang, S.-T. Lee, *ACS Nano* **2016**, 10, 7323.
[63] D. Rodríguez-Fernández, J. Langer, M. Henriksen-Lacey, L. M. Liz-Marzán, *Chemistry of Materials* **2015**, 27, 2540.
[64] D. Gkogkou, B. Schreiber, T. Shaykhutdinov, H. K. Ly, U. Kuhlmann, U. Gernert, S. Facsko, P. Hildebrandt, N. Esser, K. Hinrichs, I. M. Weidinger, T. W. H. Oates, *ACS Sensors* **2016**, 1, 318.
[65] W. Kim, J.-C. Lee, G.-J. Lee, H.-K. Park, A. Lee, S. Choi, *Analytical Chemistry* **2017**, 89, 6448.
[66] Q. Shi, K. J. Si, D. Sikdar, L. W. Yap, M. Premaratne, W. Cheng, *ACS Nano* **2016**, 10, 967.
[67] J. A. Powell, K. Venkatakrishnan, B. Tan, *Journal of Materials Chemistry B* **2016**, 4, 5713.
[68] S. Guo, X. Ren, X. Li, *Plasmonics* **2020**, 15, 591.
[69] P. Singh, T. A. F. König, A. Jaiswal, *ACS Applied Materials & Interfaces* **2018**, 10, 39380.
[70] C. Huang, C. Xu, J. Lu, Z. Li, Z. Tian, *Applied Surface Science* **2016**, 365, 291.
[71] T. Wang, Z. Zhang, F. Liao, Q. Cai, Y. Li, S.-T. Lee, M. Shao, *Scientific Reports* **2014**, 4, 4052.
[72] H.-Y. Fu, X.-Y. Lang, C. Hou, Z. Wen, Y.-F. Zhu, M. Zhao, J.-C. Li, W.-T. Zheng, Y.-B. Liu, Q. Jiang, *Journal of Materials Chemistry C* **2014**, 2, 7216.
[73] C. Zhang, S. Z. Jiang, C. Yang, C. H. Li, Y. Y. Huo, X. Y. Liu, A. H. Liu, Q. Wei, S. S. Gao, X. G. Gao, B. Y. Man, *Scientific Reports* **2016**, 6, 25243.
[74] W. Fan, Y. H. Lee, S. Pedireddy, Q. Zhang, T. Liu, X. Y. Ling, *Nanoscale* **2014**, 6, 4843.
[75] Y. Wang, K. Wang, B. Zou, T. Gao, X. Zhang, Z. Du, S. Zhou, *Journal of Materials Chemistry C* **2013**, 1, 2441.
[76] M. Zhang, Z. Zheng, H. Liu, D. Wang, T. Chen, J. Liu, Y. Wu, *ACS Omega* **2018**, 3, 5761.
[77] Z. Wang, L. Feng, D. Xiao, N. Li, Y. Li, D. Cao, Z. Shi, Z. Cui, N. Lu, *Nanoscale* **2017**, 9, 16749.
[78] S. He, W. Xie, S. Fang, X. Huang, D. Zhou, Z. Zhang, J. Du, C. Du, D. Wang, *Applied Surface Science* **2019**, 488, 707.

[79] Z. Li, S. Jiang, Y. Huo, T. Ning, A. Liu, C. Zhang, Y. He, M. Wang, C. Li, B. Man, *Nanoscale* **2018**, 10, 5897.

[80] Y. Li, X. Zhao, P. Zhang, J. Ning, J. Li, Z. Su, G. Wei, *Journal of Materials Chemistry C* **2015**, 3, 4126.

[81] Y. Shan, Y. Yang, Y. Cao, C. Fu, Z. Huang, *Nanotechnology* **2016**, 27, 145502.

[82] X. He, C. Yue, Y. Zang, J. Yin, S. Sun, J. Li, J. Kang, *Journal of Materials Chemistry A* **2013**, 1, 15010.

[83] N. D. Jayram, S. Sonia, S. Poongodi, P. S. Kumar, Y. Masuda, D. Mangalaraj, N. Ponpandian, C. Viswanathan, *Applied Surface Science* **2015**, 355, 969.

[84] X. Wang, X. Cheng, X. Yu, X. Quan, *Journal of Nanotechnology* **2018**, 2018, 9602480.

[85] M. Škrabić, M. Kosović, M. Gotić, L. Mikac, M. Ivanda, O. Gamulin, *Nanomaterials* **2019**, 9, 421.
